# Opinion Models, Election Data, and Political Theory

**DOI:** 10.3390/e26030212

**Published:** 2024-02-28

**Authors:** Matthias Gsänger, Volker Hösel, Christoph Mohamad-Klotzbach, Johannes Müller

**Affiliations:** 1 Institute of Political Science and Sociology, Julius-Maximilians-University (JMU), 97074 Würzburg, Germany; matthias.gsaenger@uni-wuerzburg.de (M.G.); christoph.mohamad-klotzbach@uni-wuerzburg.de (C.M.-K.); 2 School for Computation, Information and Technology, TU München (TUM), 80333 Munich, Germany; volker.hoesel@tum.de; 3 Institute for Computational Biology, Helmholtz Center Munich, 85764 Munich, Germany

**Keywords:** opinion dynamics, Potts models, Glauber dynamics, q-voter model, reinforcement model, weak and strong effects continuum limit, data analysis and model comparison, elections, voting behavior, interdisciplinarity

## Abstract

A unifying setup for opinion models originating in statistical physics and stochastic opinion dynamics are developed and used to analyze election data. The results are interpreted in the light of political theory. We investigate the connection between Potts (Curie–Weiss) models and stochastic opinion models in the view of the Boltzmann distribution and stochastic Glauber dynamics. We particularly find that the q-voter model can be considered as a natural extension of the Zealot model, which is adapted by Lagrangian parameters. We also discuss weak and strong effects (also called extensive and nonextensive) continuum limits for the models. The results are used to compare the Curie–Weiss model, two q-voter models (weak and strong effects), and a reinforcement model (weak effects) in explaining electoral outcomes in four western democracies (United States, Great Britain, France, and Germany). We find that particularly the weak effects models are able to fit the data (Kolmogorov–Smirnov test) where the weak effects reinforcement model performs best (AIC). Additionally, we show how the institutional structure shapes the process of opinion formation. By focusing on the dynamics of opinion formation preceding the act of voting, the models discussed in this paper give insights both into the empirical explanation of elections as such, as well as important aspects of the theory of democracy. Therefore, this paper shows the usefulness of an interdisciplinary approach in studying real world political outcomes by using mathematical models.

## 1. Introduction

The search for common compromises in a discursive social process is at the heart of all democracies. In these debates, citizens seek their standpoint on the basis of information and news, but also based on discussions with family, colleagues, and acquaintances. Opinion dynamics aims to model the basic structure of precisely this process [1,2,3].

Many opinion dynamics models are constructed on communications graphs, where the nodes represent persons, who only interact with neighboring persons [4]. Basically, there are two groups of models: either individuals are equipped with one of a finite number of opinions (typically pro and contra) or their opinions are characterized by a continuous spectrum of possibilities (typically the interval [0,1], see [5]). In particular, the models with a continuous state space are often used to investigate whether a consensus can be reached in the long run [6,7,8].

Most papers do not aim to validate their models in analyzing empirical data in a quantitative way [9]. Those papers that do address empirical data mainly focus on the dynamics of two opinions within homogeneous groups and do not use an underlying graph structure [10,11,12,13,14,15,16,17], as many interesting aspects, like different interaction patterns or phase transitions, already appear in models describing homogeneous populations and do not require interaction graphs. Here, we find a parallel in mathematical epidemiology, where it is clear that infections spread via contact graphs, but most quantitative models and methods aiming at the description and prediction of the dynamics of a real world outbreak are based on models that assume homogeneous mixing [18,19]. Also in the present work, we find that these simple models are sufficiently rich to meet the structure of empirical data. Technically, the main objective of those more empirically oriented papers is to obtain the stationary distribution of the underlying stochastic process and then to use this result in order to fit model parameters to data. In that, these papers are able discuss possible mechanisms that generate striking patterns in data [11,12,13,14,15], aim to reveal changes in communication patterns in the course of time [16], or address spatial communication distances [17]. Not only in political processes, but also in other fields such as vaccination hesitancy, opinion models contribute to an adequate description of the underlying communication mechanisms and, in that, potentially open up ways to handle (in this case) public health problems [9,20,21]. It is, however, noticeable that all these aforementioned approaches from socio-physics and socio-mathematics up to now only have a small or no echo in the social and political sciences.

In the present paper, we aim to achieve three goals: in the first part, we connect two different approaches to opinion models: one driven by stochastic dynamics and the other one originating in statistical physics; in the second part, we target a model comparison to find out which models are able to explain empirical data. The third part then discusses the usefulness of mathematical models of this type for political sciences.

For the first part of the paper: if we review the literature, we find two main approaches to describe opinion models—one approach formulates mechanisms about how people change their minds when interacting with others in the form of stochastic dynamics. In the simplest case, the voter model [22], it is assumed that a person faithfully copies the opinion of some randomly chosen other person. A slightly more refined version of this idea introduces zealots (also called stubborns or activists) who never change their mind [23,24], which results in the zealot (or noisy voter) model. In the long run, we find an equilibrium in opinion dynamics, which is termed stationary distribution. This distribution can be used to analyze empirical data.

A completely different approach originates in the statistical physics of spin systems. Herein, the dynamics of the opinions are not considered, but it is assumed that only a little is known about the state of the population, e.g., surveys could inform us about the abundance of some opinions. To express this partial knowledge appropriately, a distribution is constructed that maximizes the Shannon entropy under the constraint of the known information. The models constructed in this way are called Potts models, and the distribution is called the Boltzmann distribution [25,26]. As the stationary distribution of the dynamical opinion models, the Boltzmann distribution is also used to analyze data [26].

We connect both approaches in identifying a dynamical stochastic model (first approach), which generates a stationary distribution that coincides with a Boltzmann distribution (second approach). In this way, we associate a dynamical model with a statistical physics Potts model and vice versa. The advantage of this procedure is the construction of a unifying framework for both approaches. Based on this framework, the q-voter model [27,28,29] appears as a natural extension of the zealot model, not in view of the mechanisms modeled by the q-voter model, but in view of the underlying mathematical  structure.

We aim to apply the models to data. Election data usually aggregate information about a large number of people, as constituencies usually comprise thousands to hundreds of thousands of voters. Therefore, a continuum limit is of interest. Here, we note that the zealot model is identical to the Moran model with mutation [30], which forms the basis of population genetics [31]. In population genetics, two different diffusion limits have been established—the weak and the strong effects limit. These two approaches differ in the assumptions regarding the scaling of the parameters with respect to the population size. We investigate ways to transfer this idea to general opinion models.

In the second part, we analyze election data from the United States (US), United Kingdom (UK), France (FRA), and Germany (GER) based on four opinion dynamics models (the Curie–Weiss model, the weak and strong effects continuum limit of the q-voter model, and, additionally, the weak effects limit of the reinforcement model [21]). We use this analysis to test the models to find out to what extent they are able to describe the data not only qualitatively, but also quantitatively. The central finding is that models derived by a weak effects limit perform much better than their strong version. Potentially, these findings will be useful for future empirical studies. In the last part, we again change the focus and turn to the interpretation of our results in the light of political science. In particular, political processes and changes in social interactions potentially leave their traces in the election data, and therewith in the estimated parameters. However, this conjecture can only be confirmed based on in-depth political research. These considerations lead us to another important aspect, which is the question of the extent to which socio-mathematical models of this kind can also be a fruitful instrument as an integral part of political science, or whether the methods, objectives, and research questions of socio-mathematics and socio-physics on the one hand and political science on the other are too different.

## 2. General Structure

In this section, we first introduce the notation before introducing the two different types of models that we will investigate.

We consider a population of *N* individuals numbered 1,…,N, where each of the individuals supports either opinion A or opinion B. The opinions are coded by 1 (for A) and −1 (for B). The state space is given by $\Sigma_{2}={\left\{\pm 1\right\}}^{N}$, such that the *i*’th component $\sigma_{i}\in \left\{\pm 1\right\}$ of state $\sigma \in \Sigma_{2}$ indicates the opinion of individual *i*. We consider Cannings models [32], that is, all individuals are exchangeable and the population is homogeneous. Particularly, we do not have an interaction graph; respectively, the interaction graph is the full graph. We introduce the functions

$$n_{+}\left(\sigma \right)=\sum_{i=1}^{N}1\left(\sigma_{i}=1\right),\;n_{-}\left(\sigma \right)=N-n_{+}\left(\sigma \right),$$

which count the supporters of opinion A (function $n_{+}\left(\sigma \right)$) and the supporters of opinion B (function $n_{-}\left(\sigma \right)$), respectively. Our knowledge on the opinion distribution in the population will be expressed by random measures on $\Sigma_{2}$. Due to the assumption of Cannings models, the random measures, necessarily, are invariant with respect to the permutations of individuals: two states $\sigma^{1},\sigma^{2}\in \Sigma_{2}$ with $n_{+}\left(\sigma^{1}\right)=n_{+}\left(\sigma^{2}\right)$ have the same probability.

That is, any random measure $Q:\Sigma_{2}\to \left[0,1\right]$ describing the state of the population induces a random measure *P* on the state space $V_{N}:=\left\{0,..,N\right\}$, which indicates the number of opinion-A supporters. Let $\sigma^{\left(k\right)}\in \Sigma_{2}$ be a given state with $n_{+}\left(\sigma^{\left(k\right)}\right)=k$; then, for combinatorial reasons:

(1) P(k)=NkQ(σ(k)).

We will find out later that the binomial coefficient, which appears here for symmetry reasons, plays a distinct role in the theory developed below.

In the next two sections, we introduce two very different ways used in the literature to construct random measures for the opinion state of the population, that is, on $\Sigma_{2}$, respectively, $V_{N}$. The first approach is based on stochastic processes, while the second is statistical in design. We should keep in mind that—due to the symmetry discussed—every rate and every function used to define the models can be constructed in such a way that it depends on $\sigma \in \Sigma_{2}$ only via $n_{\pm }\left(\sigma \right)$ and the population size *N*.

### 2.1. Opinion Process

A stochastic opinion process is a $\Sigma_{2}$-valued Markov process ${\hat{\sigma }}_{t}$, where single persons reconsider their opinion at rate ν. There is a certain probability that this person indeed changes her mind. These probabilities depend on the opinion distribution in the population; as mentioned above, this dependency is established via $n_{\pm }\left(\sigma \right)$ and not via the fine-structure of the state (which individual is an A- and which individual is a B-supporter). For mathematical convenience, but without loss of generality, we assume that the rate for $\sigma_{i}i$ to switch from −1 to 1 is a function of $n_{+}\left(\sigma \right)$ and *N*, while the rate to switch from 1 to −1 depends on $n_{-}\left(\sigma \right)$ and *N*,

(2a) $$\begin{array}{ccc} \sigma_{i}=-1\to \;\;\;1 & \mathrm{at}\;\mathrm{rate} & \nu f^{+}\left(n_{+}\left(\sigma \right);N\right) \end{array}$$

(2b) $$\begin{array}{ccc} \sigma_{i}=\;\;\;1\to -1 & \mathrm{at}\;\mathrm{rate} & \nu f^{-}\left(n_{-}\left(\sigma \right);N\right). \end{array}$$

To obtain a feeling for which terms to use for $f^{\pm }$, we can look at the voter model. Herein, ν represents the rate at which a person reconsiders her opinion and interacts with some randomly chosen person in the population (with so-called selfing, that is, the person might also choose herself). The functions $f^{\pm }\left(n_{\pm }\left(\sigma \right)\right)=n_{\pm }\left(\sigma \right)/N$ simply specify the probability of interacting with a person of the other opinion. Below, we also consider other examples, where the probability to change the mind is slightly more involved, but the overall structure of the terms will be similar. All other entries in state σ are not affected by a flip of the *i*’th person’s opinion.

As mentioned above, the $\Sigma_{2}$-valued process ${\hat{\sigma }}_{t}$ induces a $V_{N}=\left\{0,..,N\right\}$-valued process $X_{t}$ via $X_{t}=n_{+}\left({\hat{\sigma }}_{t}\right)$. The transition rates of $X_{t}$ are given by

(3a) $$\begin{array}{ccc} X_{t}=k\to k+1 & \mathrm{at}\;\mathrm{rate} & \nu \left(N-k\right)\;f^{+}\left(k;N\right) \end{array}$$

(3b) $$\begin{array}{ccc} X_{t}=k\to k-1 & \mathrm{at}\;\mathrm{rate} & \nu k\;\;\;\;f^{-}\left(N-k;N\right). \end{array}$$

We call a Markov process with transition rates given in this form an opinion process. It is straightforward to determine the stationary distribution of an opinion process if we have no absorbing states. As we aim at a specific notation that parallels the usual notation of Potts models, we derive the stationary distribution step by step. In what follows, we suppress the dependency of $f^{\pm }$ on *N*.

**Proposition** **1** (stationary distribution)**.** *Assume $f^{\pm }\left(k\right)>0$ for $k\in V_{N}=\left\{0,\ldots ,N\right\}$. Furthermore, let $F_{\pm }:V_{N}\to R$ be defined by*

(4) $$\begin{array}{c} F_{\pm }\left(0\right)=0,\;F_{\pm }\left(k\right)=\frac{1}{k}\sum_{l=0}^{k-1}\ln\left(f^{\pm }\left(l\right)\right)\;for\;k\in V_{N}\setminus \left\{0\right\}. \end{array}$$

*Denoting the probability of state $k\in V_{N}$ in the stationary distribution by $p_{k}$, we have*

(5) pk=e−H˜(k)Z˜,H˜(k)=−ln(Nk)−kF+(k)−(N−k)F−(N−k),Z˜=∑k=0Ne−H˜(k).

**Proof.**  The detailed balance equation for the stationary distribution $p_{k}=P\left(X=k\right)$ yields

(6) $$\begin{array}{c} p_{k}\;k\;f^{-}\left(N-k\right)=p_{k-1}\left(N-k+1\right)\;f^{+}\left(k-1\right). \end{array}$$

Hence,

pk=p0∏l=1k(N−l+1)f+(l−1)lf−(N−l)=p0∏l=1kN−l+1k!∏l=1kf+(l−1)f−(N−l)=p0Nk∏l=1kf+(l−1)f−(N−l)=p0∏l=1Nf−(N−l)Nk∏l=1kf+(l−1)∏l=k+1Nf−(N−l)=p0∏l=1Nf−(l)Nk∏l=1kf+(l−1)∏l=1N−kf−(l−1)=CNkexp∑l=0k−1ln(f+(l))+∑l=0N−k−1ln(f−(l)),

where *C* is determined by $\sum_{k=0}^{N}p_{k}=1$. Together with the definition of $F_{\pm }$, $\tilde{H}\left(k\right)$, and $\tilde{Z}$, this formula proves the proposition.    □

Interestingly, the corresponding stationary distribution on $\Sigma_{2}$ can be written as

(7) $$\begin{array}{c} Q\left(\sigma \right)=Z^{-1}\;\exp\left\{n_{+}\left(\sigma \right)\;F_{+}\left(n_{+}\left(\sigma \right)\right)+n_{-}\left(\sigma \right)\;F_{-}\left(n_{-}\left(\sigma \right)\right)\right\}=Z^{-1}\;\prod_{i=1}^{N}\exp\left(\;F_{\sigma_{i}}\left(n_{\sigma_{i}}\left(\sigma \right)\right)\;\right), \end{array}$$

where $F_{\sigma_{i}}=F_{+}$ if $\sigma_{i}=1$, and $F_{\sigma_{i}}=F_{-}$ if $\sigma_{i}=-1$; this is similar for $n_{\sigma_{i}}$. Each individual has an independent contribution $\exp(F_{\sigma_{i}}\left(.\right))$ to the probability of state σ, where $F_{\pm }\left(.\right)$ depend on the global statistics of the state via $n_{\pm }\left(\sigma \right)$. That is, $F_{\sigma_{i}}\left(n_{\sigma_{i}}\left(\sigma \right)\right)$ can be regarded as the environment of individual *i*, which determines the probability of the opinion that individual *i* has adopted. It is, furthermore, interesting to observe that the population size *N* does not explicitly appear in the expression $\exp(F_{\sigma_{i}}\left(n_{\sigma_{i}}\left(\sigma \right)\right))$. The number of the opposite-opinion-supporters only comes in indirectly, as $n_{+}\left(\sigma \right)$ and $n_{-}\left(\sigma \right)$ add up to the given population size *N*.

For obvious reasons, we call the functions $F_{\pm }\left(.\right)$ the environmental conditions, or simply the environments, of the opinion process.

We have a degree of freedom in Equation (5). We might add a real constant *A* in the exponent, pk=Z˜−1Nke−H˜(k)+A. Then, $\tilde{Z}$ is still defined as the normalizing constant, guaranteeing that $\sum_{k=0}^{N}p_{k}=1$. Thus, also in $\tilde{Z}$, the term $e^{A}$ appears such that *A* cancels out and does not affect the value of $p_{k}$. Below, in the definition of the Boltzmann distribution, we will find a similar invariance. This invariance will be used later to eliminate singularities appearing in Section 3.2, where we investigate the large population limit with weak effects.

For now, let us discuss the freedom given by this invariance more in detail and, in particular, explore the implication for the choice of the environments. If we replace $F_{\pm }\left(k\right)$ by ${\tilde{F}}_{\pm }\left(k\right)$,

$$\begin{array}{c} {\tilde{F}}_{\pm }\left(k\right)=F_{\pm }\left(k\right)+U_{\pm }\left(k\right)/k,\;\;k=1,\ldots ,N, \end{array}$$

where we choose $U_{\pm }\left(0\right)=0$ in accordance with $F_{\pm }\left(0\right)=0$ and require $U_{\pm }\left(k\right)$ to satisfy

$$\begin{array}{c} U_{+}\left(k\right)+U_{-}\left(N-k\right)=A,\;\;k\in V_{N}. \end{array}$$

Then, the stationary distribution $p_{k}$ is not affected. We now go backward and determine transition rates $k{\tilde{f}}^{\pm }\left(N-k\right)$ that produce the new environments. Here, we use $\ln\left({\tilde{f}}^{\pm }\left(k\right)\right)=\left(k+1\right)\;{\tilde{F}}_{\pm }\left(k+1\right)-k\;{\tilde{F}}_{\pm }\left(k\right)=\ln\left(f^{\pm }\left(k\right)\right)+U_{\pm }\left(k+1\right)-U_{\pm }\left(k\right)$ such that

$${\tilde{f}}^{\pm }\left(k\right)=f^{\pm }\left(k\right)\frac{e^{U_{\pm }\left(k+1\right)}}{e^{U_{\pm }\left(k\right)}}.$$

Since $U_{-}\left(N-k\right)=A-U_{+}\left(k\right)$, we have

$${\tilde{f}}^{-}\left(N-k\right)=f^{-}\left(N-k\right)\frac{e^{U_{-}\left(N-k+1\right)}}{e^{U_{-}\left(N-k\right)}}=f^{-}\left(N-k\right)\frac{e^{A-U_{+}\left(k-1\right)}}{e^{A-U_{+}\left(k\right)}}=f^{-}\left(N-k\right)\frac{e^{U_{+}\left(k\right)}}{e^{U_{+}\left(k-1\right)}}.$$

The function $U_{+}$ indeed cancels out in the detailed balance of Equation (6).

Please note that we are free to choose $U_{+}\left(k\right)$ (with the understanding that $U_{+}\left(0\right)=0$). Next, we defined $A=U_{+}\left(N\right)$. Then, $U_{-}\left(k\right)$ is already determined via $U_{-}\left(N-k\right)=A-U_{+}\left(k\right)$ for $k\in V_{N}$. In particular, the choice $A=U_{+}\left(N\right)$ is equivalent with the requirement $U_{-}\left(0\right)=0$ such that *A* is not free. The choice $A=U_{+}\left(N\right)$ is unique. All in all, the freedom we have is completely characterized by the choice of $U_{+}$.

**Corollary** **1.** *The rates $k\;f^{\pm }\left(k\right)$ (or, alternatively, the environments $F_{\pm }\left(k\right)=\frac{1}{k}\sum_{l=0}^{k-1}\ln\left(f^{\pm }\left(l\right)\right)$) determine the stationary distribution of an opinion process. The set of all opinion processes with stationary distributions coinciding with that given distribution is characterized by*

(8) $$\begin{array}{c} {\tilde{f}}^{\pm }\left(k\right)=f^{\pm }\left(k\right)\frac{e^{U_{\pm }\left(k+1\right)}}{e^{U_{\pm }\left(k\right)}}\;resp.\;{\tilde{F}}_{\pm }\left(k\right)=F_{\pm }\left(k\right)+U_{\pm }\left(k\right)/k, \end{array}$$

*where $U_{\pm }$ are functions satisfying $U_{+}\left(0\right)=U_{-}\left(0\right)=0$ and*

(9) $$\begin{array}{c} U_{+}\left(k\right)+U_{-}\left(N-k\right)=A\;for\;k\in V_{N}\;and\;A=U_{+}\left(N\right)\in R. \end{array}$$

### 2.2. Potts Machinery

Next, we introduce Potts models, where we again consider Potts models only on a full graph. Potts models on a full graph are often termed mean-field or Curie–Weiss models. In agreement with the literature, we return for the moment to the individual-based formulation of the opinion model, that is, we use $\Sigma_{2}$ as a state space.

We do not know the state of the population. The knowledge we assume to have are the results of some polls. For example, we could observe/measure the fraction of individuals with opinion +1. Consequently, we will know the expected number of persons in state 1, that is, $n_{+}\left(.\right)$. As the Potts models originate in physics, we follow the tradition and call these polls “observations” and, accordingly, the function $n_{+}\left(.\right)$ an “observable”.

In general, observables are defined as functions $\hat{F}:\Sigma_{2}\to R$ without further restrictions. Another example of an observable that is often used is the number of pairs with identical opinions minus the number of pairs with different opinions,

$$\hat{F}\left(\sigma \right)=\sum_{k=1}^{N-1}\sum_{l=k+1}^{N}\sigma_{k}\sigma_{l}.$$

This observable incorporates information about correlations in the population.

Let us assume that we have *m* observables ${\hat{F}}_{1},\ldots ,{\hat{F}}_{m}$. Our knowledge about the state of the population is restricted to the knowledge of ${\hat{f}}_{l}:={\hat{F}}_{l}\left(\sigma \right)$, l=1,…,m. We represent our knowledge, and, particularly, the absence of complete knowledge about the state, in the form of a random measure *Q* on $\Sigma_{2}$. We, of course, require that $E\left({\hat{F}}_{l}\left(.\right)\right)={\hat{f}}_{l}$ such that *Q* does express our partial knowledge appropriately. However, there are many random measures that will satisfy this requirement. We express this lack of complete knowledge by uniquely defining *Q* as the random measure that maximizes the Shannon entropy $S\left(Q\right)=-\sum_{\sigma \in \Sigma_{2}}Q\left(\sigma \right)\ln\left(Q\left(\sigma \right)\right)$, under the constraints $E\left({\hat{F}}_{l}\left(.\right)\right)={\hat{f}}_{l}$. The random measure Q(.) we construct in this way is called the Boltzmann distribution.

We restrict ourselves to Cannings models such that the observables ${\hat{F}}_{l}$ only depend on $\Sigma_{2}$ via $n_{\pm }\left(\sigma \right)$ and thus can be defined as maps ${\hat{F}}_{l}:V_{N}\to R$. Also, the Boltzmann distribution can be defined directly on $V_{N}$ via P(n+(σ))=Nn+(σ)Q(σ). As $Q\left(\sigma \right)=Q\left(\tilde{\sigma }\right)$ if $n_{+}\left(\sigma \right)=n_{+}\left(\tilde{\sigma }\right)$, this formula defines *P* consistently. However, as the lack of knowledge still concerns the state of the population in $\Sigma_{2}$, we do not use the original Shannon entropy for *P*, but measure the entropy for *P* by the entropy for the associated measure *Q* on $\Sigma_{2}$,

SΣ2(P):=−∑σ∈Σ2Q(σ)ln(Q(σ))=−∑k∈VNNkP(k)Nk−1ln(P(k)Nk−1)=−∑k∈VNP(k)ln(P(k))−ln(Nk).

**Proposition** **2.** *Let m∈N denote the number of observables and ${\hat{F}}_{l}:V_{N}\to R$, l∈{1,..,m} the observables themselves. The Boltzmann distribution is the distribution $P:V_{N}\to R_{+}$ that maximizes the Shannon entropy SΣ2(P)=−∑k∈VNP(k)ln(P(k))−ln(Nk) under the constraint $E\left({\hat{F}}_{l}\left(.\right)\right)={\hat{f}}_{l}\in R$. If the Boltzmann distribution P exists, then*

(10) P(k)=e−H(k)Z,H(k)=−ln(Nk)−∑l=1mλlF^l(k),Z=∑k∈VNe−H(k),

*where $\lambda_{l}$, l∈{1,..,m} are Langrange multipliers.*

**Proof.**  The proof consists of a short computation (see, e.g., [25,26]), based on the standard Lagrangian approach for the maximization of S(P) under the constraints $E\left({\hat{F}}_{l}\left(.\right)\right)=\sum_{k\in V_{N}}{\hat{F}}_{l}\left(k\right)\;P\left(k\right)={\hat{f}}_{l}$, l=1,…,m, and $\sum_{k\in V_{N}}\;P\left(k\right)=1$. Let P(.) denote the set of probabilities P(k)∈[0,1] for $k\in V_{N}=\left\{0,\ldots ,N\right\}$. We determine all values P(k) by maximizing the function

$$L\left(P\left(.\right),\lambda_{1},..,\lambda_{m+1}\right)=S_{\Sigma_{2}}\left(P\right)+\sum_{l=1}^{m}\lambda_{l}\left(\;E\left({\hat{F}}_{l}\left(.\right)\right)-{\hat{f}}_{l}\;\right)+\lambda_{m+1}\left(\;\sum_{k\in V_{N}}P\left(k\right)\;-\;1\;\right).$$

Fix $\tilde{k}\in V_{N}$. If we equate the derivative of *L* with respect to $P(\tilde{k})$ to zero, we find

0=∂L(P(.))∂P(k˜)=−ln(P(k˜))+ln(Nk˜)−1+∑l=1mλlF^l(k˜)+λm+1

and $P\left(\tilde{k}\right)=e^{-H(\tilde{k})}\;\;e^{\lambda_{m+1}-1}$. We obtain $e^{\lambda_{m+1}-1}=1/Z$ by the condition that the probabilities add up to 1, that is, $\sum_{k\in V_{N}}P\left(k\right)\;=\;1$.   □

**Remark** **1.** *(a) In accordance with the literature, the Lagrangian multipliers $\lambda_{1},\ldots ,\lambda_{m}$ are not specified to actually determine a Boltzmann distribution that indeed satisfies $E\left({\hat{F}}_{l}\left(.\right)\right)={\hat{f}}_{l}\in R$, but instead, the Lagrangian multipliers are from now on considered as parameters of the Boltzmann distribution.*

*(b) We note that additive constants in the Hamiltonian do not affect the stationary distribution, as these constants appear in a multiplicative way in numerator $e^{-H(k)}$ as well as in the denominator Z of the stationary distribution. We make use of this observation below to get rid of singularities appearing in the weak effects limit.*

The Curie–Weiss model in sensu stricto is defined by observables that are polynomials of the second order, as we will discuss in Section 4.1. For the time being (Section 2.3 and Section 3), we allow for more general functions as observables, as this is necessary to obtain and utilize a connection between the Curie–Weiss models and the opinion processes, which we discuss next.

### 2.3. Connection between Opinion Processes and the Curie–Weiss Model

An opinion process has a stationary distribution, and a Potts model a Boltzmann distribution. In order to connect opinion processes and Potts models, we ask which conditions the observables (Potts models), respectively, the environments (opinion processes) need to satisfy such that the stationary distribution of an opinion model coincides with the Boltzmann distribution. If we combine Propositions 1 and 2, we find the following corollary.

**Corollary** **2.** *The stationary distribution of an opinion model with population size N and environment $F_{\pm }:V_{N}\to R$ and the Boltzmann distribution for observables ${\hat{F}}_{\pm }:V_{N}\to R$ coincide, if*

(11) $$\begin{array}{c} {\hat{F}}_{\pm }\left(k\right)=k\;F_{\pm }\left(k\right)\;and\;\lambda_{\pm }=1. \end{array}$$

Note that this corollary only states sufficient but not necessary conditions. Corollary 1 allows, for example, for constructing more observables that generate the same distribution. Considering the settings of this corollary for general $\lambda_{\pm }\in R$, we observe

$$\lambda_{\pm }{\hat{F}}_{\pm }\left(k\right)=\lambda_{\pm }k\;F_{\pm }\left(k\right)=\sum_{l=0}^{k-1}\ln\left(\;\;f^{\pm }{\left(l\right)}^{\lambda_{\pm }}\;\right).$$

Therefore, a Boltzmann distribution with observables derived from environments of an opinion process is, for general $\lambda_{\pm }\in R$, the stationary distribution of the opinion process with transition rates

(12a) $$\begin{array}{ccc} X_{t}\to X_{t}+1 & \mathrm{at}\;\mathrm{rate} & \nu \;\left(N-k\right)\;f^{+}{\left(k\right)}^{\lambda_{+}} \end{array}$$

(12b) $$\begin{array}{ccc} X_{t}\to X_{t}-1 & \mathrm{at}\;\mathrm{rate} & \nu \;\;\;\;k\;\;\;f^{-}{\left(N-k\right)}^{\lambda_{-}}. \end{array}$$

We call this family of opinion models the Glauber family for the given observables/environments. Please note that the standard Glauber dynamics for the spin up/spin down mean-field Ising model [25] utilized the freedom discussed in Corollary 1 in choosing a non-trivial function $U_{+}\left(k\right)$.

## 3. Large Population Limits

Below, we consider applications of opinion processes to data, where, often, the population size is in the magnitude of $N\approx 10^{5}$. Therefore, a continuity limit is of interest. As above, we might consider the dynamics (opinion process) and work out a diffusion limit, or we might focus on the Boltzmann distribution, and consider a continuum limit for that distribution. The interesting point is to understand the connection between the two resulting objects.

Herein, we note that the rates $f^{\pm }$, the corresponding environments $F_{\pm }$, respectively, and the observables ${\hat{F}}_{\pm }$ incorporate parameters. Two different assumptions about the dependencies of these parameters on the population size *N* (which itself is a parameter) lead to sensible continuum limits for N→∞: one is the strong effects limit and the other the weak effects limit, as introduced for the Moran model as part of population genetics [33,34,35] (where the Moran model with mutation is mathematically identical to the zealot model). In physics, the terms “extensive” and “nonextensive” models are used to describe the same idea [36,37].

To explain the difference between strong and weak effects, let us focus on the zealot model. In the strong effects models, the number of zealots is assumed to increase proportional with the population size. In that, the probability for a person to interact with a zealot is independent of the population size, and zealots clearly will affect the invariant distribution and also in the limiting case, N→∞.

In the weak effects setting, however, the number of zealots is constant and independent of the population size. A person’s probability to interact with a zealot tends toward zero if the population size tends toward infinity. The effects of zealots become weaker and weaker if *N* increases, which is why these models are termed “weak effects models”. Mobilia, indeed, asked in his paper [23]: “Does a single zealot affect an infinite group of voters?” Though it sounds unlikely that this single zealot has some noteworthy effect on a huge population, it turns out that this is the case.

We define the weak/strong effects limit assumptions in a mathematically precise way in Section 3.1 and Section 3.2 below (please do not confuse the weak effects limit, which refers to the scaling of the parameters, with a weak limit, which refers to the topology of convergence).

We assume throughout the current section that $f^{\pm }$ and ${\hat{F}}_{\pm }$ depend on k/N for $k\in V_{N}$, that is, on the sharing of an opinion x=k/N,

$$\begin{array}{ccc} f^{\pm }\left(k;N\right)\; & \mathrm{is}\;\mathrm{replaced}\;\mathrm{by} & \;f^{\pm }\left(k/N;N\right)\;k\in V_{N},\\ {\hat{F}}_{\pm }\left(k;N\right)\; & \mathrm{is}\;\mathrm{replaced}\;\mathrm{by} & \;{\hat{F}}_{\pm }\left(k/N;N\right)\;k\in V_{N}, \end{array}$$

where

$$f^{\pm }:\left[0,1\right]\times N\to R,\;{\hat{F}}_{\pm }:\left[0,1\right]\times N\to R$$

such that $f^{\pm }\left(x;N\right)$, $F_{\pm }\left(x;N\right)$ are well-defined for all x∈[0,1]. Since the observables are defined via $f^{\pm }$, we rewrite them separately; see Section 3.1 below.

For given *N*, the old and the new scaling are mathematically equivalent. However, if we aim at a limit N→∞, the new scaling is a reasonable and very helpful assumption that the most commonly used models actually fulfill.

### 3.1. Strong Effects Limit

In the strong effects limit, we assume that $f^{\pm }\left(x;N\right)$ approximate functions $f^{\pm }\left(x\right)\in C^{2}$ for N→∞ such that

(13) $$\begin{array}{c} \lim_{N\to \infty }f^{\pm }\left(x;N\right)=f^{\pm }\left(x\right)\mathrm{in}\;C^{2}\left[0,1\right] \end{array}$$

is well-defined. For clarity of notation, we write the limits of rate functions, environments, and observables for N→∞ in bold. To adapt the definition region of the associated environments from the discrete state space $V_{N}$ to the continuous state space x∈[0,1], we re-define the environments as

$$F_{\pm }:\left[0,1\right]\times N\to R,\;F_{\pm }\left(x;N\right):=\frac{1}{N\;x}\sum_{l=0}^{\lfloor N\;x\rfloor -1}\ln\left(f^{\pm }\left(l/N;N\right)\right).$$

For x=k/N, $k\in V_{N}$, we recover the environments as defined in Proposition 1. Therewith, the environments also satisfy a proper limit for N→∞,

(14) $$\begin{array}{c} F_{\pm }\left(x\right):=\lim_{N\to \infty }F_{\pm }\left(x;N\right)=\lim_{N\to \infty }\frac{1}{x}\sum_{l=0}^{\lfloor N\;x\rfloor -1}\ln\left(f^{\pm }\left(l/N;N\right)\right)\frac{1}{N}=x^{-1}\;\int_{0}^{x}\ln\left(f^{\pm }\left(y\right)\right)\;dy. \end{array}$$

We also introduce a limit for the observables. Here, a certain subtlety appears: we have $\hat{F}\left(k/N;N\right)=kF\left(k/N;N\right)$. As x=k/N, this formula becomes $\hat{F}\left(k/N;N\right)=N\;xF\left(x;N\right)$. In leading order, the observables are O(N). We thus scale them by 1/N and define

(15) $$\begin{array}{c} {\hat{F}}_{\pm }\left(x\right):=\lim_{N\to \infty }\frac{1}{N}\hat{F}\left(k/N;N\right)=xF_{\pm }\left(x\right)=\int_{0}^{x}\ln\left(f^{\pm }\left(y\right)\right)\;dy. \end{array}$$

We emphasize at this point that when using ${\hat{F}}_{\pm }\left(x\right)$ below, we need to take the scale 1/N, introduced at this point, into account.

To obtain the behavior of the opinion process under the strong effects limit, we briefly sketch the Kramers Moyal [38] expansion of the model, which is—as usual—truncated at the second order to obtain a Fokker–Planck (or Kolmogorov forward) equation.

**Proposition** **3.** *The Kramers Moyal expansion up to the second order for the Glauber family with limiting observables ${\hat{F}}_{\pm }\left(x\right)=\int_{0}^{x}\ln\left(f^{\pm }\left(y\right)\right)\;dy$ is given by*

(16) $$\begin{array}{ccc} \partial_{t}u\left(x,t\right) & = & -\nu \;\partial_{x}\left(\;\left(\left(1-x\right)\;f^{+}{\left(x\right)}^{\lambda_{+}}-x\;f^{-}{\left(1-x\right)}^{\lambda_{-}}\;\right)\;u\left(x,t\right)\right)\\ & & +\frac{\nu }{2N}\;\partial_{xx}\left(\;\left(\left(1-x\right)\;f^{+}{\left(x\right)}^{\lambda_{+}}+x\;f^{-}{\left(1-x\right)}^{\lambda_{-}}\;\right)\;u\left(x,t\right)\right). \end{array}$$

**Proof.**  We start with the master equations

$$\begin{array}{ccc} {\dot{p}}_{k} & = & -\nu \;\left(\left(N-k\right)f^{+}{\left(k/N;N\right)}^{\lambda_{+}}+k\;f^{-}{\left(1-k/N;N\right)}^{\lambda_{-}}\right)p_{k}\\ & & +\nu \left(N-\left(k-1\right)\right)f^{+}{\left(\;\left(k-1\right)/N\right)}^{\lambda_{+}}p_{k-1}+\nu \left(k+1\right)f^{-}{\left(1-\left(k+1\right)/N\right)}^{\lambda_{-}}p_{k+1}. \end{array}$$

If we now assume that $p_{k}\approx h\;u\left(x,t\right)$ for some smooth probability density u(x,t), where x=kh and h=1/N, we have $\partial_{t}u\left(x,t\right)\approx {\dot{p}}_{k}$, and

$$\begin{array}{ccc} \partial_{t}h\;u\left(x,t\right) & = & -\nu \;h^{-1}\left(\left(1-x\right)f^{+}{\left(x;N\right)}^{\lambda_{+}}+xf^{-}{\left(1-x;N\right)}^{\lambda_{-}}\right)\;u\left(x,t\right)\\ & & +\nu \;h^{-1}\;\left(1-x-h\right)\;f^{+}{\left(x-h;N\right)}^{\lambda_{+}}u\left(x-h,t\right)\\ & & +\nu \;h^{-1}\;\left(x+h\right)\;f^{-}{\left(1-x-h;N\right)}^{\lambda_{-}}u\left(x+h,t\right). \end{array}$$

The Taylor expansion of the last two terms up to the second order neglecting the error term and using the limit of $f^{\pm }\left(\;.\;;N\right)$ for N→∞ yields the Kramers Moyal expansion.    □

Next, we turn to the Boltzmann distribution. In the continuum limit, we will denote the Boltzmann distribution (and later the stationary distribution of a limiting stochastic opinion process as well) by φ(x).

**Proposition** **4.** *The Boltzmann distribution for observables ${\hat{F}}_{\pm }\left(x\right)=\int_{0}^{x}\ln\left(f^{\pm }\left(y\right)\right)\;dy$ and large N is given in leading order by the Hamiltonian*

(17) $$\begin{array}{c} H\left(x\right)=N\left(-H_{2}\left(x\right)-\lambda_{+}\int_{0}^{x}\ln\left(f^{+}\left(y\right)\right)\;dy-\lambda_{-}\int_{0}^{1-x}\ln\left(f^{-}\left(y\right)\right)\;dy\right), \end{array}$$

*where $H_{2}\left(x\right)=-x\ln\left(x\right)-\left(1-x\right)ln\left(1-x\right)$ is the binary entropy. The limiting Boltzmann distribution reads $\varphi \left(x\right)=e^{-H(x)}/Z\left(x\right)$, where $Z\left(x\right)=\int_{0}^{1}e^{-H(x)}\;dx$.*

The result is a consequence of Equation (10), the scale of ${\hat{F}}_{\pm }$ with respect to *N* introduced in (15), and the well-known approximation of the binomial coefficient by means of the binary entropy

(18) ln(Nn)=NH2(n/N)−12ln(2πN)−12ln(x(1−x))+O(N−1).

The connection between the stationary distributions of the Kramers Moyal expansion Equation (16) and the stationary distribution Equation (17) is not clear; though they are derived from the associated Potts models and opinion processes, they look rather different. The next proposition clarifies the connection.

**Proposition** **5.** *Assume that N is large. Let $f\left(x\right)=\left(1-x\right)\;f^{+}{\left(x\right)}^{\lambda_{+}}-x\;f^{-}{\left(1-x\right)}^{\lambda_{-}}$, and assume f(μ)=0, $f^{\prime }\left(\mu \right)<0$ for some μ∈(0,1). Then, the local normal approximation of the Boltzmann distribution (17) coincides with the stationary distribution of the Ornstein–Uhlenbeck approximation of the Kramers Moyal expansion (16) at x=μ in leading order in N.*

**Proof.**  We first investigate the Boltzmann distribution. Since

$$\frac{H^{\prime }\left(\mu \right)}{N}=\ln\left(\mu \right)-\ln\left(1-\mu \right)-\lambda_{+}\ln\left(f^{+}\left(\mu \right)\right)+\lambda_{-}\ln\left(f^{-}\left(1-\mu \right)\right)=\ln\left(\frac{\mu \;f^{-}{\left(1-\mu \right)}^{\lambda_{-}}}{\left(1-\mu \right)\;f^{+}{\left(\mu \right)}^{\lambda_{+}}}\right)=0,$$

we have a critical point of H(x) at x=μ. The second derivative reads, again using f(μ)=0,

$$\begin{array}{ccc} & & \frac{H^{\prime \prime }\left(\mu \right)}{N}\\ & = & \frac{\left(1-\mu \right)\;f^{+}{\left(\mu \right)}^{\lambda_{+}}}{\mu \;f^{-}{\left(1-\mu \right)}^{\lambda_{-}}}\;\frac{\left(1-\mu \right)\;f^{+}{\left(\mu \right)}^{\lambda_{+}}\frac{d}{d\mu }\left(\mu \;f^{-}{\left(1-\mu \right)}^{\lambda_{-}}\right)-\mu \;f^{-}{\left(1-\mu \right)}^{\lambda_{-}}\frac{d}{d\mu }\left(\left(1-\mu \right)\;f^{+}{\left(\mu \right)}^{\lambda_{+}}\right)}{{\left(\left(1-\mu \right)\;f^{+}{\left(\mu \right)}^{\lambda_{+}}\right)}^{2}}\\ & = & \frac{\frac{d}{d\mu }\left(\mu \;f^{-}{\left(1-\mu \right)}^{\lambda_{-}}\right)-\frac{d}{d\mu }\left(\left(1-\mu \right)\;f^{+}{\left(\mu \right)}^{\lambda_{+}}\right)}{\left(1-\mu \right)\;f^{+}{\left(\mu \right)}^{\lambda_{+}}}=\frac{-f^{\prime }\left(\mu \right)}{\left(1-\mu \right)\;f^{+}{\left(\mu \right)}^{\lambda_{+}}}=\frac{|f^{\prime }\left(\mu \right)|}{\left(1-\mu \right)\;f^{+}{\left(\mu \right)}^{\lambda_{+}}}. \end{array}$$

Hence, $H\left(x\right)=H\left(\mu \right)+\frac{1}{2}{\left(x-\mu \right)}^{2}H^{\prime \prime }\left(\mu \right)+O\left({\left(x-\mu \right)}^{3}\right)$. Locally, at x=μ, the stationary distribution $e^{-H(x)}/Z$ behaves as $N(\mu ,\sigma^{2})$ with

$$\sigma^{2}=H^{\prime \prime }{\left(\mu \right)}^{-1}=\frac{\left(1-\mu \right)\;f^{+}{\left(\mu \right)}^{\lambda_{+}}}{N\;|f^{\prime }\left(\mu \right)|}=\frac{\mu \;f^{-}{\left(\mu \right)}^{\lambda_{-}}}{N\;|f^{\prime }\left(\mu \right)|}.$$

Next, we proceed to the stationary distribution based on the Kramers Moyal expansion (16). The leading order terms of the linearization of the drift and the noise term at x=μ yield the Ornstein–Uhlenbeck approximation

(19) $$\begin{array}{ccc} \partial_{t}u\left(x,t\right) & = & -\nu \;\partial_{x}\left(\;\left(x-\mu \right)f^{\prime }\left(\mu \right)\;u\left(x,t\right)\right)+\frac{\nu }{N}\;\partial_{xx}\left(\;\left(1-\mu \right)\;f^{+}{\left(\mu \right)}^{\lambda_{+}}\;u\left(x,t\right)\right)\\ & = & \partial_{x}\left\{\nu \;\left(\;\left(x-\mu \right)f^{\prime }\left(\mu \right)\;u\left(x,t\right)\right)+\frac{\nu }{N}\;\partial_{x}\left(\;\left(1-\mu \right)\;f^{+}{\left(\mu \right)}^{\lambda_{+}}\;u\left(x,t\right)\right)\right\} \end{array}$$

where we used $\left(1-\mu \right)\;f^{+}{\left(\mu \right)}^{\lambda_{+}}=\mu \;f^{-}{\left(1-\mu \right)}^{\lambda_{-}}$ in the noise term. In order to identify the stationary distribution, we substitute u(x,t)=φ(x) with $\varphi \left(x\right)=e^{-a{\left(x-\mu \right)}^{2}}$ into the term bracketed with curly brackets,

$$\begin{array}{ccc} & & \nu \;\left(\;\left(x-\mu \right)f^{\prime }\left(\mu \right)\;e^{a{\left(x-\mu \right)}^{2}}\right)+\frac{\nu }{N}\;\partial_{x}\left(\;\left(1-\mu \right)\;f^{+}{\left(\mu \right)}^{\lambda_{+}}\;e^{-a{\left(x-\mu \right)}^{2}}\right)\\ & = & \left\{f^{\prime }\left(\mu \right)\;-2a\frac{\nu }{N}\;\left(1-\mu \right)\;f^{+}{\left(\mu \right)}^{\lambda_{+}}\;\right\}\left(x-\mu \right)\;e^{-a{\left(x-\mu \right)}^{2}}. \end{array}$$

This term becomes zero and, therewith, φ(x) an invariant measure, if $a^{-1}=2\frac{\left(1-\mu \right)\;f^{+}{\left(\mu \right)}^{\lambda_{+}}}{N\;|f^{\prime }\left(\mu \right)|}$. Therefore, the stationary distribution of this approximate Fokker–Planck equation is a normal distribution $N(\mu ,\sigma^{2})$, where

$$\sigma^{2}=\frac{\left(1-\mu \right)\;f^{+}{\left(\mu \right)}^{\lambda_{+}}}{N\;|f^{\prime }\left(\mu \right)|}=\frac{\mu \;f^{-}{\left(1-\mu \right)}^{\lambda_{-}}}{N\;|f^{\prime }\left(\mu \right)|}.$$

   □

For large *N*, the stationary distribution will be concentrated close to μ and, hence, in the relevant regions both stationary distributions, that of Equation (19) and of Equation (17), coincide. In that, we consider the Kramers Moyal expansion (16) as the Glauber dynamics of the Boltzmann distribution.

### 3.2. Weak Effects Limit

The idea of the weak effects limit is to take a simple reference model as a basis and to perturb this model in such a way that for N→∞, the transition rates converge back to that of the reference model. For good reasons, as we will find out later, we use the voter model as the reference model. In the voter model, at rate ν, a person copies the opinion of a randomly chosen person in the population (inclusive “selfing”—that does mean that the focal person might by chance copy her opinion from herself). Therewith,

(20a) $$\begin{array}{ccc} X_{t}=k\to k+1 & \mathrm{at}\;\mathrm{rate} & \nu \left(N-k\right)\;f_{voter}^{+}\left(k/N\right)=\nu \left(N-k\right)\;\frac{k}{N} \end{array}$$

(20b) $$\begin{array}{ccc} X_{t}=k\to k-1 & \mathrm{at}\;\mathrm{rate} & \nu k\;\;\;\;f_{voter}^{-}\left(1-k/N\right)=\nu k\;\;\;\;\frac{N-k}{N}, \end{array}$$

and hence, $f_{voter}^{\pm }\left(x\right)=x$. For the weak effects limit, we allow for rates $f^{\pm }\left(x;N\right)$, which depend on *N*, as long as $\lim_{N\to \infty }f^{\pm }\left(x;N\right)=f_{voter}^{\pm }\left(x\right)$. Also, the Lagrangian parameters are allowed to depend on *N*, $\lambda_{\pm }=\lambda_{\pm }\left(N\right)$, and the paradigm of weak effects requires again $\lim_{N\to \infty }f^{\pm }{\left(x;N\right)}^{\lambda_{\pm }\left(N\right)}=f_{voter}^{\pm }\left(x\right)$. Therefore, the expansion of $f^{\pm }$ with respect to 1/N has $f_{voter}^{\pm }\left(x\right)$ as the zero order term and some arbitrary (well-behaved) function $g^{\pm }\left(x\right)$ as the first order coefficient. Similarly, the zero order term of the expansion of $\lambda^{\pm }\left(N\right)$ is 1, while the first order terms are some parameters $\kappa^{\pm }\in R$, which we are free to choose. All in all, the Glauber family suited for the weak effects limit assumes the form

(21) $$\begin{array}{c} f^{\pm }{\left(k/N;N\right)}^{\lambda_{\pm }\left(N\right)}={\left(f_{voter}^{\pm }\left(k/N\right)+\frac{1}{N}g^{\pm }\left(k/N\right)+O\left(N^{-2}\right)\right)}^{1+\kappa_{\pm }/N}. \end{array}$$

As we will see, for the weak effects limit, we not only assume the appropriate scaling of the parameters, but also rescale time. In that, even for N→∞, we still obtain a non-trivial limiting process and do not simply return to the voter model.

As a consequence, we need to re-consider the large population limit conducted in (14) and (15) more in detail and also pay attention to the terms of order $O(N^{-1})$.

**Proposition** **6.** *Consider the observables for the opinion process defined by* (21),

$${\hat{F}}_{\pm }\left(x;N\right)=\sum_{l=0}^{\lfloor N\;x\rfloor -1}\ln\left(\frac{l}{N}+\frac{1}{N}g^{\pm }\left(l/N\right)+O\left(N^{-2}\right)\right)\;\frac{1}{N}.$$

*With the definition*

(22) $$\begin{array}{c} G^{\pm }\left(x\right)=\int^{x}g^{\pm }\left(y\right)/y\;dy, \end{array}$$

*the leading order terms of the expansion of ${\hat{F}}_{\pm }\left(x;N\right)$ in 1/N reads*

$${\hat{F}}_{\pm }\left(x;N\right)=x\;\left(\ln\left(x\right)-1\right)-\frac{1}{2\;N}\ln\left(x\right)+\frac{1}{N}G^{\pm }\left(x\right)+O\left(N^{-2}\right).$$

**Proof.**  We first rewrite the sum such that it extends to ⌊Nx⌋ instead of ⌊Nx⌋−1,

$$\begin{array}{ccc} {\hat{F}}_{\pm }\left(x;N\right) & = & \sum_{l=0}^{\lfloor N\;x\rfloor -1}\ln\left(\frac{l}{N}+\frac{1}{N}g^{\pm }\left(l/N\right)+O\left(N^{-2}\right)\right)\;\frac{1}{N}\\ & = & \sum_{l=0}^{\left\lfloor N\;x\right\rfloor }\ln\left(\frac{l}{N}+\frac{1}{N}g^{\pm }\left(l/N\right)+O\left(N^{-2}\right)\right)\;\frac{1}{N}\\ & & \;-\ln\left(\frac{\left\lfloor N\;x\right\rfloor }{N}+\frac{1}{N}g^{\pm }\left(\left\lfloor N\;x\right\rfloor /N\right)+O\left(N^{-2}\right)\right)\;\frac{1}{N}. \end{array}$$

Next, we replace the sum by an integral. Here, we take the Euler–McLaurin correction terms into account in the step from sum to integral. Furthermore, we note that an additive constant in the Hamiltonian does not change the stationary distribution. Instead of $\sum_{l=0}^{\left\lfloor Nx\right\rfloor }\left(\ldots \right)$, we can change the lower starting value to any value independent on *x* and might, e.g., consider $\sum_{l=\lfloor N/2\rfloor }^{\left\lfloor Nx\right\rfloor }\left(\ldots \right)$ instead. Only the upper bound of the sum matters; we only need an anti-derivative. To express this fact, we skip the lower bound of the integral and proceed (where the first equal sign has to be interpreted with the knowledge that we did drop some irrelevant term):

$$\begin{array}{ccc} {\hat{F}}_{\pm }\left(x;N\right) & = & \int^{\left\lfloor N\;x\right\rfloor }\ln\left(\frac{l}{N}+\frac{1}{N}g^{\pm }\left(l/N\right)+O\left(N^{-2}\right)\right)\;dl\;\frac{1}{N}\\ & & \;-\frac{1}{2}\ln\left(\frac{\left\lfloor N\;x\right\rfloor }{N}+\frac{1}{N}g^{\pm }\left(\left(\left\lfloor N\;x\right\rfloor \right)/N\right)+O\left(N^{-2}\right)\right)\;\frac{1}{N}+O\left(N^{-2}\right)\\ & = & \int^{x}\ln\left(y+\frac{1}{N}g^{\pm }\left(y\right)\right)\;dy-\frac{1}{2}\ln\left(x+\frac{1}{N}g^{\pm }\left(x\right)\right)\;\frac{1}{N}+O\left(N^{-2}\right)\\ & = & \int^{x}\ln\left(y+\frac{1}{N}g^{\pm }\left(y\right)\right)\;dy-\frac{\ln(x)}{2N}-\frac{1}{2}\ln\left(1+\frac{1}{N}g^{\pm }\left(x\right)/x\right)\;\frac{1}{N}+O\left(N^{-2}\right)\\ & = & \frac{-1}{2\;N}\ln\left(x\right)+\int^{x}\ln\left(y\;\;\left\{1+\frac{1}{Ny}g^{\pm }\left(y\right)\right\}\right)\;dy+O\left(N^{-2}\right)\\ & = & x\left(\ln\left(x\right)-1\right)-\frac{1}{2\;N}\ln\left(x\right)+\int^{x}\ln\left(1+\frac{1}{Ny}g^{\pm }\left(y\right)\right)\;dy+O\left(N^{-2}\right). \end{array}$$

Note that $\lim_{N\to \infty }\int^{x}\ln\left(1+\frac{1}{y\;N}g^{\pm }\left(y\right)\right)\;dy=0$ (in the sense that 0 is a possible limiting anti-derivative) such that this integral only contributes to terms of order $O(N^{-1})$ or higher. We introduce

$$G^{\pm }\left(x\right)=\lim_{N\to \infty }N\int^{x}\ln\left(1+\frac{1}{y\;N}g^{\pm }\left(y\right)\right)\;dy=\int^{x}g^{\pm }\left(y\right)/y\;dy$$

and obtain the result.    □

Therewith, we are in the position to establish the following proposition.

**Proposition** **7.** *Assume that the functions $f^{\pm }\left(x\right)$ scale with N as described above,*

$$f^{\pm }\left(x;N\right)=\left(x+\frac{1}{N}g^{\pm }\left(x\right)+O\left(N^{-2}\right)\right)$$

*and scale the Lagrangian parameters by $\lambda_{\pm }=1+\kappa_{\pm }/N$. Then, in leading order, the Hamiltonian reads*

(23) $$\begin{array}{ccc} H(x) & = & \ln\left(x\left(1-x\right)\right)-G^{+}\left(x\right)-G^{-}\left(1-x\right)-\kappa_{+}\;\zeta \left(x\right)-\kappa_{-}\;\zeta \left(1-x\right) \end{array}$$

*with ζ(x)=−x(1−ln(x)).*

**Proof.**  Recall $H_{2}\left(x\right)=-x\ln\left(x\right)-\left(1-x\right)\ln\left(1-x\right)$. With the scaling $\lambda_{\pm }=1+\kappa_{\pm }/N$, we obtain

$$\begin{array}{ccc} & & \lambda_{+}{\hat{F}}_{+}\left(x;N\right)+\lambda_{-}{\hat{F}}_{-}\left(x;N\right)\\ & = & -N\;H_{2}\left(x\right)-N-\frac{1}{2}\ln\left(x\left(1-x\right)\right)+\kappa_{+}x\ln\left(x\right)+\kappa_{-}\left(1-x\right)\ln\left(1-x\right)\\ & & -\kappa_{+}x-\kappa_{-}\left(1-x\right)+G^{+}\left(x\right)+G^{-}\left(1-x\right)+O\left(N^{-1}\right). \end{array}$$

We use the approximation of the binomial coefficient (18) to obtain the result with H(x)=−ln(NNx)−λ+F^+(x;N)−λ−F^−(x;N), where we drop terms independent of *x* and terms of a higher order in $N^{-1}$.    □

It is remarkable and typical for the weak effects scaling that the Hamiltonian and, in that, also the Boltzmann distribution become independent of *N*. We now turn to the underlying opinion process and discuss the Kramers Moyal expansion under the scaling assumed.

**Proposition** **8.** *The Kramers Moyal expansion of the Glauber family under the weak effects scaling in rescaled time T=νt/N is given by*

(24) $$\begin{array}{ccc} u_{T}\left(x,T\right) & = & -\partial_{x}\left((\left(1-x\right)g^{+}\left(x\right)-xg^{-}\left(x\right)+\kappa_{+}\left(1-x\right)\ln\left(x\right)+\kappa_{-}x\ln\left(1-x\right))\;u\left(x,T\right)\right)\\ & & +\partial_{xx}\left(x(1-x)\;u(x,T)\right). \end{array}$$

**Proof.**  The Glauber family with the weak effects scaling is defined by

$$f^{\pm }\left(k/N\right)={\left(\frac{k}{N}+\frac{1}{N}g^{+}\left(k/N\right)+O\left(N^{-2}\right)\right)}^{1+\kappa_{\pm }/N},$$

while the rate $X_{t}=k\to k+1$ reads $\nu \left(1-x\right)f^{+}{\left(..\right)|}_{k=x\;N}$ and that for $X_{t}=k\to k-1$ is $\nu \;x\;f^{-}{\left(..\right)|}_{k=(1-x)\;N}$. The drift term becomes in leading order

$$\begin{array}{ccc} & & \nu \left(1-x\right)\;{\left(x+\frac{1}{N}g^{+}\left(x\right)+O\left(N^{-2}\right)\right)}^{1+\kappa_{+}/N}-\nu x{\left(\left(1-x\right)+\frac{1}{N}g^{-}\left(1-x\right)+O\left(N^{-2}\right)\right)}^{1+\kappa_{-}/N}\\ & = & \frac{\nu }{N}\left(\left(1-x\right)g^{+}\left(x\right)-xg^{-}\left(x\right)+\kappa_{+}\left(1-x\right)x\ln\left(x\right)+\kappa_{-}x\left(1-x\right)\ln\left(1-x\right)\right)+h.o.t. \end{array}$$

(where h.o.t. is a placeholder for higher order terms), and the coefficient of the noise term becomes in leading order

$$\begin{array}{ccc} & & \nu \left(1-x\right)\;{\left(x+\frac{1}{N}g^{+}\left(x\right)+O\left(N^{-2}\right)\right)}^{1+\kappa_{+}/N}+\nu x{\left(\left(1-x\right)+\frac{1}{N}g^{+}\left(1-x\right)+O\left(N^{-2}\right)\right)}^{1+\kappa_{-}/N}\\ & = & 2\;\nu \;x(1-x)+h.o.t. \end{array}$$

If we rescale time T=νt/N, we obtain the result.   □

The Kramers Moyal expansion in rescaled time becomes, as the Hamiltonian, independent of *N*.

**Proposition** **9.** *The stationary distribution of the Kramers Moyal expansion is identical with the Boltzmann distribution φ(x)=exp(−H(x))/Z, where the Hamiltonian H(x) is given in* (23).

**Proof.**  We plug the Boltzmann distribution into the right-hand side of the Kramers Moyal expansion. Thereto, we note that

$$\varphi^{\prime }\left(x\right)=-\varphi \left(x\right)\;H^{\prime }\left(x\right)=-\left(\frac{1}{x}-\frac{1}{1-x}-{\left(G^{+}\right)}^{\prime }\left(x\right)+{\left(G^{-}\right)}^{\prime }\left(1-x\right)-\kappa_{+}\ln\left(x\right)-\kappa_{-}\ln\left(1-x\right)\right)\varphi \left(x\right).$$

Furthermore, $\frac{d}{dx}G^{\pm }\left(x\right)=g^{\pm }\left(x\right)/x$ such that

$$\begin{array}{ccc} & & \partial_{x}\left(x(1-x)\varphi (x)\right)=-x\varphi \left(x\right)+\left(1-x\right)\varphi \left(x\right)+x\left(1-x\right)\varphi^{\prime }\left(x\right)\\ & = & \left(\left(1-x\right)g^{+}\left(x\right)-xg^{-}\left(x\right)+x\left(1-x\right)\kappa_{+}\ln\left(x\right)+\kappa_{-}x\left(1-x\right)\ln\left(1-x\right)\right)\varphi \left(x\right). \end{array}$$

If we take the derivative of this equation with respect to *x*, we indeed find that φ(x) is a stationary solution of Equation (24).   □

**Remark** **2.** *For the weak effects limit, we have chosen the voter model with $f^{\pm }\left(x\right)=x$ as the reference model. If N→∞, the rates of the model at hand converge back to this model. This choice looks, at first glance, arbitrary. It is, however, up to the freedom characterized in Corollary 1, a unique choice: the binary entropy $H_{2}\left(x\right)$ generates in the Hamiltonian terms Nxln(x) and N(1−x)ln(1−x). For a weak effects limit to exist, these terms of order O(N) need to be balanced and annihilated by the environments of the reference model, which already forces the reference model to be the voter model (or some model, which is, according to Corollary 1, equivalent to the voter model).*

## 4. Four Models: Curie–Weiss, Weak and Strong q-Voter Model, and Reinforcement

We use the framework introduced above to briefly introduce four opinion models we intend to apply to data.

### 4.1. Curie–Weiss Model

To introduce the classical Curie–Weiss model, we start with the Potts machinery. Recall that the central ingredients are observables, that is, functions ${\hat{F}}_{\pm }:V_{N}\to R$, which form constraints when determining the random measure maximizing the Shannon entropy. Perhaps the most simple, non-trivial case is given by observables, which are polynomials of the second order,

$$F_{\pm }\left(k/N;N\right)=a_{\pm }k+b_{\pm }k^{2}/N=N{\left(a_{\pm }\;x\;+b_{\pm }\;x^{2}\;\right)}_{x=k/N}.$$

We do not need a zero order term, as additive constants in the Hamiltonian do not influence the Boltzmann distribution. Furthermore, we scale the quadratic term by 1/N to balance the squared terms in case of large *N*. With this setting, the Hamilton defined in (10) reads

H(x)=−ln(NNx)−λ+F+(x;N)−λ−F−(1−x;N)=−ln(NNx)−Nλ+b+x2+λ−b−(1−x)2+λ+a+x+λ−a−(1−x).

We might rewrite the quadratic terms as

$$\lambda_{+}b_{+}x^{2}+\lambda_{-}b_{-}{\left(1-x\right)}^{2}=\frac{\left(\lambda_{+}b_{+}+\lambda_{-}b_{-}\right)}{2}\left(x^{2}+{\left(1-x\right)}^{2}\right)+\left(\lambda_{+}b_{+}-\lambda_{-}b_{-}\right)x+\frac{\lambda_{-}b_{-}-\lambda_{+}b_{+}}{2}$$

We define $J=\lambda_{+}b_{+}+\lambda_{-}b_{-}$ and $h_{\pm }$ appropriately (e.g., $h_{+}=\lambda_{+}b_{+}-\lambda_{-}b_{-}$). Furthermore, for historical reasons, we introduce $h_{-}=0$ and drop terms independent of *x*. Therewith, we obtain

(25) H(x)=−ln(NxN)−NJ2x2+(1−x)2+h+x+h−(1−x),

which is the standard form of the model on the state space $V_{N}$ [25]. We can still reduce the number of parameters from four ($N,J,h_{+},h_{-}$) to three (N,J,h) with $h=h_{+}-h_{-}$ as the additive constant, which, apparently, can again be dropped.

Last, we check the existence of the strong and the weak effects limit. The strong effects limit can be derived trivially, simply by replacing the binomial coefficient by $NH_{2}\left(x\right)$, cf. (18),

$$H\left(x\right)=-N\left(H_{2}\left(x\right)+\frac{J}{2}\left(x^{2}+{\left(1-x\right)}^{2}\right)+h_{+}x+h_{-}\left(1-x\right)\right).$$

The binary entropy introduces logarithmic terms of order *N* into the Hamiltonian H(x). As the observables of the Curie–Weiss model consist of polynomial terms, they cannot cancel these logarithmic terms such that the Hamiltonian always incorporates nontrivial terms of order O(N). In the proper weak effects limit, however, terms of this order are not present. Thus, a weak effects limit for the Curie–Weiss model is not possible (cf. Remark 2). This will be different for the other models we shall discuss next.

### 4.2. Two Flavors of the q-Voter Model

For the q-voter model, we do not start with the stationary distribution but the transition rates. Perhaps the most simple extension of the voter model where no opinion can die out is the zealot model, where we have $N^{\pm }$ zealots for the opinion ±1. Palombi and Toti ([24] p. 337) call zealots “stubborn agents [*…*] who never change political preference”. In our case, zealots are not real persons but represent sources of information that stand for a specific opinion. These could be politicians, friends, newspapers, or social media channels. As in the voter model, individuals copy their opinion from a randomly chosen person, now also from the zealots, which leads to

$$f^{\pm }\left(k;N\right)=\frac{N^{\pm }+k}{N+N^{-}+N^{+}}.$$

The corresponding Glauber family is given by

(26a) $$\begin{array}{ccc} X_{t}=k\to k+1 & \mathrm{at}\;\mathrm{rate} & \nu \left(N-k\right)\;{\left(\;\frac{N^{+}+k}{N+N^{-}+N^{+}}\right)}^{\lambda_{+}} \end{array}$$

(26b) $$\begin{array}{ccc} X_{t}=k\to k-1 & \mathrm{at}\;\mathrm{rate} & \nu k\;{\left(\;\frac{N^{-}+\left(N-k\right)}{N+N^{-}+N^{+}}\right)}^{\lambda_{-}}. \end{array}$$

For $\lambda_{+}=\lambda_{-}>1$, this is the q-voter model for a homogeneous population [27]. In the case of $\lambda_{\pm }=1$, we are back in the zealot model: a person simply copies the opinion of another person or a zealot. If $\lambda_{\pm }>1$, the model can be interpreted as follows: the person will ask $\lambda_{\pm }$ other persons for their opinion and will only change her opinion if all these other persons have the identical opinion.

#### 4.2.1. q-Voter Model—Strong Effects

We consider the strong effects limit: if $N^{\pm }=\eta^{\pm }\;N$, that is, if the number of zealots scales linearly with the population size where $\eta^{\pm }$ are the proportionality constants,

$$f^{\pm }\left(x;N\right)=\frac{N\;\eta^{\pm }\;+N\;x}{N+N\;\eta^{-}+N\;\eta^{+}}=\frac{\eta^{\pm }+x}{1+\eta^{-}+\eta^{+}},$$

such that $f^{\pm }\left(x;N\right)$ becomes independent of *N*, and $f^{\pm }\left(x;N\right)\equiv f^{\pm }\left(x\right)$. Therewith, the limiting observables are given by

$${\hat{F}}_{\pm }\left(x\right)=\int_{0}^{x}\ln\left(\frac{\eta^{\pm }+y}{1+\eta^{-}+\eta^{+}}\right)\;dy=\left(x+\eta^{\pm }\right)\;\ln\left(\eta^{\pm }+x\right)-x-x\ln\left(1+\eta^{-}+\eta^{+}\right)+C$$

where *C* is a constant. The stationary distribution in the strong effects limit is given by $\varphi \left(x\right)=Z^{-1}\exp\left(-H\left(x\right)\right)$, where

(27) $$\begin{array}{ccc} H(x) & = & -N\;(H_{2}\left(x\right)+\lambda_{+}\left[\left(x+\eta^{+}\right)\;\ln\left(\eta^{+}+x\right)-x\left(1+\ln\left(1+\eta^{-}+\eta^{+}\right)\right)\right]\\ & & \;\;+\lambda_{-}\left[\left(\left(1-x\right)+\eta^{-}\right)\;\ln\left(\eta^{-}+\left(1-x\right)\right)-\left(1-x\right)\left(1+\ln\left(1+\eta^{-}+\eta^{+}\right)\right)\right]). \end{array}$$

In the last formula, we again made use of the fact that we are allowed to drop constant terms from the Hamiltonian.

#### 4.2.2. q-Voter Model—Weak Effects

We now go into the weak effects limit for the q-voter model. The basis is the zealot model with $f^{\pm }\left(k;N\right)=\frac{N^{\pm }+k}{N+N^{-}+N^{-}}.$ For the weak effects limit, the number of zealots $N^{\pm }$ does not scale with the population size, and in that, zealots become rare if *N* becomes large. We can choose the time units, and in this, we have a degree of freedom in the form of a multiplicative positive constant. This freedom can be used to replace the original denominator $N+N^{-}+N^{-}$ by *N* and work with

$$f^{\pm }\left(x;N\right)=x+\frac{1}{N}\;g^{\pm }\left(x\right),\;g^{\pm }\left(x\right)\equiv N^{\pm }.$$

That is, for our particular choice, the functions $g^{\pm }\left(x\right)$ are independent of *x*. Recall that we also expand the Lagrangian parameters in terms of *N* and write $\lambda_{\pm }=1+\kappa_{\pm }/N$.

To obtain the weak effects limit, we note that $G^{\pm }\left(x\right)=\int^{x}g^{\pm }\left(y\right)/y\;dy=\ln\left(x\right)\;N^{\pm }$ and obtain the Hamiltonian

$$H\left(x\right)=-\ln\left(x\right)\left(N^{+}-1\right)-\ln\left(1-x\right)\left(N^{-}-1\right)-\kappa_{+}x\ln\left(x\right)-\kappa_{-}\left(1-x\right)\ln\left(1-x\right)+\left(\kappa_{+}-\kappa_{-}\right)\;x.$$

with the corresponding stationary distribution

(28) $$\begin{array}{c} \varphi \left(x\right)=C\;x^{N^{+}-1}\;{\left(1-x\right)}^{N^{-}-1}\;e^{-\kappa_{+}x\left(1-\ln\left(x\right)\right)}\;\;e^{-\kappa_{-}\left(1-x\right)\left(1-\ln\left(1-x\right)\right)}. \end{array}$$

The stationary distribution becomes a beta distribution in the case of the zealot model ($\kappa_{\pm }=0$); this result was first derived in the context of population genetics, where the zealot model is termed the Moran model ([33] page 108). The extension to the weak effects limit of the Glauber family presented here is novel.

### 4.3. Reinforcement Model—Weak Effects

We add one more model, which also allows for a strong as well as weak effects limit and which is, as the q-voter model, also a descendant from the zealot model: the reinforcement model [21]. The idea of the reinforcement model is to express the psychological mechanisms that lead to filter bubbles and echo chambers: several kinds of cognitive biases let individuals communicate with persons of the opposite opinion with less awareness than with individuals of their own opinion. Some interactions with the opposite group are ignored. In that, the effective size of the opposite group is reduced by a factor $\theta_{\pm }\in \left(0,1\right]$. The zealot model is described by

$$f^{\pm }\left(k/N;N\right)=\frac{\theta_{\pm }\left(N^{\pm }+k\right)}{N^{\pm }+N-k+\theta_{\pm }\left(N^{\mp }+k\right)}.$$

We focus on the weak effects limit and hence keep (as in the weak limit of the q-voter model) $N^{\pm }$ independent of *N*. Furthermore, we choose $\theta_{\pm }=1-\vartheta_{\pm }/N$ such that we return to the voter model if *N* becomes large; the Lagrangian parameters are taken to be $\lambda_{\pm }=1$ and are not scaled. It turns out that the computations assume a simpler form (additive constants will vanish below in the first order term of the expansion) if we use a trivial time scale such that a multiplicative term $N^{+}/N+1+N^{-}/N$ appears in the rates,

$$f^{\pm }\left(k/N;N\right)=\left(N^{+}/N+1+N^{-}/N\right)\;\frac{\left(1-\vartheta_{\pm }/N\right)\left(N^{\pm }+k\right)}{N^{\pm }+N-k+\left(1-\vartheta_{\pm }/N\right)\;\left(N^{\mp }+k\right)}.$$

Therewith, we obtain the expansion of the rates with respect to 1/N,

$$f^{\pm }\left(x;N\right)=x+\frac{1}{N}g^{\pm }\left(x\right)+O\left(N^{-2}\right),\;g^{\pm }\left(x\right)=\vartheta_{\pm }x^{2}-\vartheta_{\pm }x+N^{\pm }.$$

Consequently, we obtain

$$\begin{array}{ccc} G^{\pm }\left(x\right) & = & \int^{x}\frac{g^{\pm }\left(y\right)}{y}\;dy=\frac{1}{2}\vartheta_{\pm }x^{2}-\vartheta_{\pm }x+N^{\pm }\;\ln\left(x\right)=-\frac{1}{2}\vartheta_{\pm }\left(2x-x^{2}\right)+N^{\pm }\;\ln\left(x\right) \end{array}$$

and the stationary distribution (we use Equation (23) with $\kappa_{\pm }=0$)

(29) $$\begin{array}{ccc} \varphi (x) & = & C\;\;x^{N^{+}-1}\;{\left(1-x\right)}^{N^{-}-1}\;\;e^{-\vartheta_{+}x\left(2-x\right)/2}\;\;e^{-\vartheta_{-}\left(1-x\right)\left(2-\left(1-x\right)\right)/2}. \end{array}$$

**Figure 1:**
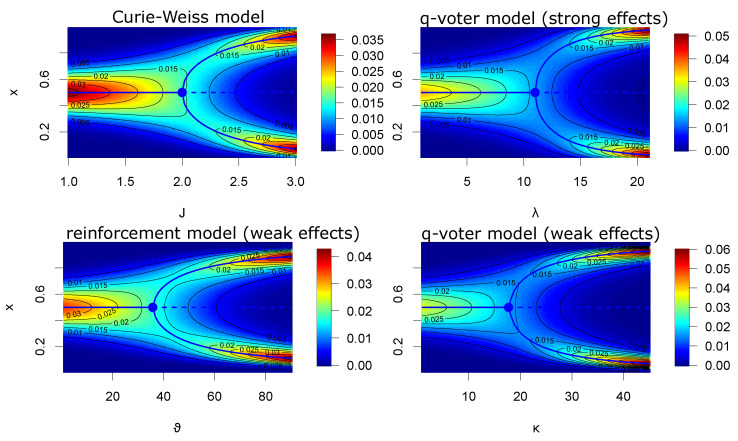
Phase transitions of the four models in the symmetric case. For a given parameter (x-axis), the density of the distribution is indicated (by the heat and contour plot) over *x* (y-axis). The blue lines indicate local maxima (solid lines) and local minima (dashed line) if the parameter (given at the x-axis) is fixed, while the dot marks the phase transition. (Curie–Weiss: $h_{\pm }=0$; strong q-voter: $\eta_{\pm }=5$ and N=20; weak q-voter: $N^{\pm }=10$, $\kappa_{\pm }=\kappa$; weak reinforcement: $N^{\pm }=10$, $\vartheta_{\pm }=\vartheta$).

If we compare the stationary distribution of the weak effects q-voter model and the weak effects reinforcement model, we find a striking similarity: in both cases, the measure is an adaptation of the beta distribution, $\varphi \left(x\right)=C\;\;x^{N^{+}-1}\;{\left(1-x\right)}^{N^{-}-1}\;\;e^{a_{+}\zeta \left(x\right)}\;\;e^{a_{-}\zeta \left(1-x\right)}$ where $a_{\pm }=\kappa_{\pm }$ and ζ(x)=−x(1−ln(x)) in the q-voter case, while $a_{\pm }=\vartheta_{\pm }/2$ and ζ(x)=−x(2−x) in the reinforcement case. As both functions ζ(x) resemble each other in that ζ(0)=0, ζ(1)=1, and both are convex, we expect very similar behavior for the two models if we take $\vartheta_{\pm }=2\kappa_{\pm }$ (also inspect Figure 1).

As a last remark, we note that we do allow, in the weak q-voter model and the weak reinforcement model, not only for positive parameter values $\kappa_{\pm }$ and $\vartheta_{\pm }$, but also for negative values. While positive values for these parameters lead to filter bubbles and echo chambers (a person hesitates to change her mind), negative values have the interpretation that the person iss open-minded and pays particular attention to the opposite opinion. In the case of the functioning of democracies and their institutions, open-minded people are much more preferable because they are easier to find compromises for solving political problems.

### 4.4. Model Behavior

We will not go deeper into the analysis (which can be found, e.g., for the Curie–Weiss model in [25,26], for the q-voter model in the strong effects limit in [27], and for the reinforcement model in [21]), but simply refer to Figure 1, which shows that all four models undergo a phase transition if the coupling between the individuals is sufficiently large. We also emphasize that the models based on the voter and zealot model (which do not allow for phase transitions per se), the two kinds of q-voter model, and the reinforcement model exhibit phase transitions. The mechanisms modifying the effects of zealots target in/outgroup communication. If in/outgroup communication is sufficiently strong, a bimodal distribution appears via a phase transition. Also, the behavior under non-symmetric conditions (parameters) leads to similar behavior of all four models.

It is interesting to note that the Curie–Weiss and the strong effects q-voter model incorporate the population size *N* explicitly, while the (weak effects limit of) the q-voter and the reinforcement model become independent of *N*. Usually, in applications, *N* is very large, and if we naively take *N* to the population size, the variance generated by the model is much smaller than the variance that is present in empirical data. The way out is to assume that individuals cluster together and to estimate an effective population size $N=N_{eff}$ along with the other parameters, which, of course, is slightly dubious but pragmatic [26]. The weak effects models elegantly circumvent this difficulty.

## 5. Data Analysis

We use data from four different Western democracies that represent different types of government and electoral systems (see [39] pp. 145–161, 271, and [40] and Table 1 for further details). Concerning the governmental system, we have one presidential (US), one semi-presidential (France), and two parliamentary systems (UK, Germany). In the case of the electoral systems, we have two majority systems with a first-past-the-post design and relative majority (US, UK) and one majority system in France with a two-round system and absolute majority. In Germany, we have a mixed member proportional system that combines both a first-past-the-post vote and proportional representation. Finally, for each country, we study different numbers of elections (US: six presidential elections, 2000–2020; UK: 20 parliamentary elections, 1945–2019; FRA: second round of five presidential elections, 2002–2022; GER: two parliamentary elections, 2017–2021; the data sources are indicated in the data availability statement).

Such a design is useful in understanding how the models used can explain the dynamics in different institutional settings with different political cultures and in varying periods of time. This comparative approach gives us more information about the functioning of the mechanisms in different contexts (e.g., [12,13]) and contributes to the existing research often based on single-case studies (e.g., [24,41]). In the analysis, we consider each election district as an i.i.d. repetition of the election. Herein, we obviously reduce the complexity of the data by neglecting social co-factors and spatial effects. In that, we obtain an empirical distribution of vote shares and can use a maximum likelihood estimator. Please find the technical details, particularly the algorithm used to perform the maximum-likelihood estimation, in Appendix A.

The present approach to data analysis is based on a steady state assumption, that is, the opinion formation process is assumed to be approximately in equilibrium. If there is a huge shift in the vote share of a candidate or party in recent times, this steady state assumption might not be met. The tables with estimates, p-values for the Kolmogorov–Smirnov test, and AICs can be found in Appendix A.

**Figure 2:**
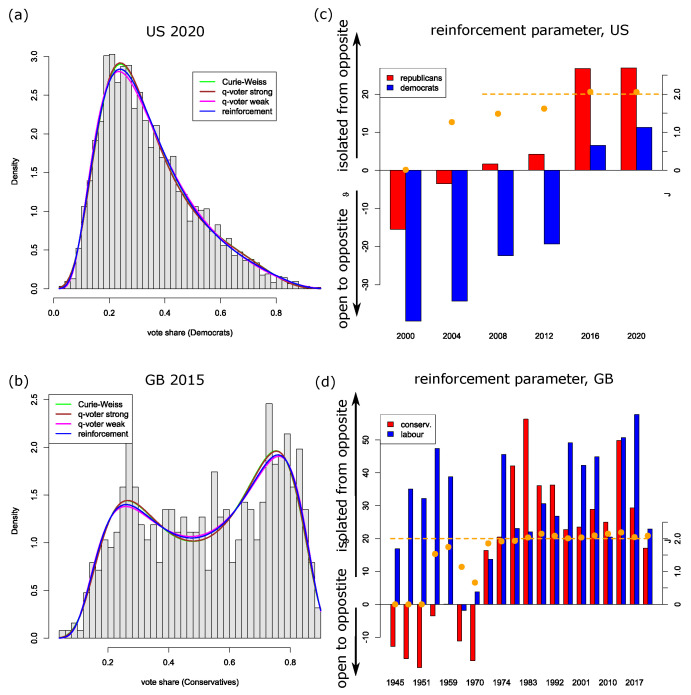
(**a**,**b**): Histograms of the vote shares for (**a**) the presidential US elections in 2020 and (**b**) the parliamentary elections in 2015 in UK, together with the probability density for the four models. (**c**,**d**): Bars indicate the reinforcement parameters $\vartheta_{\pm }$ for (**c**) Democrats and Republicans, and (**d**) the conservatives and the Labour Party (left axis); orange bullets indicate parameter *J* of the Curie–Weiss model (right axis), while the horizontal dashed orange line indicates the threshold for the phase transition of that model for h=0.

**Figure A1:**
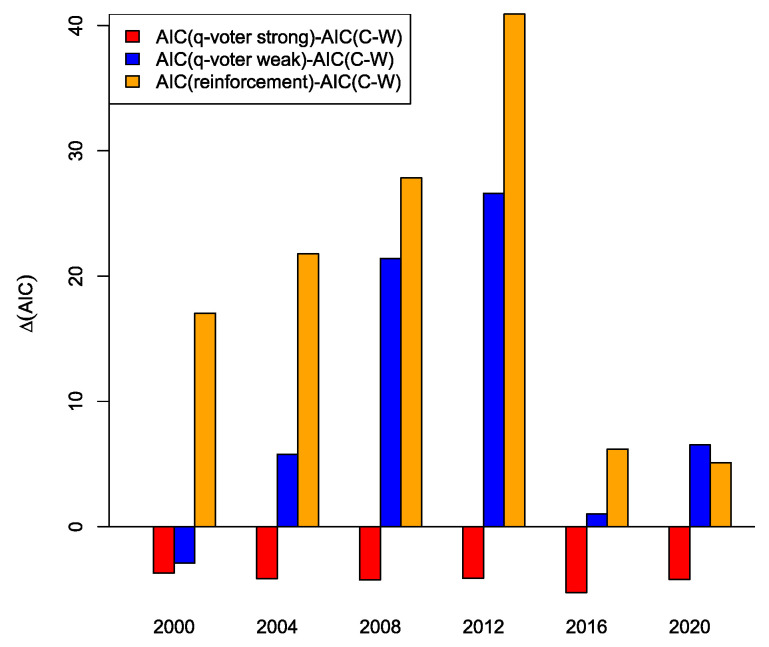
Comparison of the models’ AICs for the US.

United States data: The densities of the four models (Curie–Weiss, weak and strong q-voter model, and reinforcement model) are rather similar (Figure 2a), and the Kolmogorov–Smirnov tests also resemble each other (see table in Appendix A.1). In the year 2000 only, the p-values of this test were small (between 0.018 and 0.04); in that year, apart from the Democrats and the Republicans, the green candidate did win a small but reasonable fraction of votes such that the dichotomous models might not be completely suited. In 2016, we also have about four percent of third-party votes, but at that point, the models fit better. Maybe this is due to the fact that polarization in the American society has already grown during these past 16 years. The AIC always selects the (weak) reinforcement model as the best-suited model, but in the year 2020, the weak q-voter model fit best. However, in this year, the reinforcement model and the weak q-voter model are very close. The strong models always perform worse (Figure A1). We clearly find a trend in the parameters, which shows that the model moves in time more and more towards a phase transition.

United Kingdom data: The trend in the reinforcement parameters/coupling is particularly interesting (Figure 2d). As can be clearly visualized in the empirical and the estimated distributions for the election from 2015, the UK indeed became super-critical. We observe a bimodal distribution in 2015. It is most interesting to see that the theoretical prediction of possible phase transitions is realized in the UK.

France data: Particularly in 2022, the empirical distribution of vote shares is skewed. The strong effects models have difficulties dealing with this result, while the weak effects models are more flexible; in particular, the reinforcement model still performs well.

**Figure 3:**
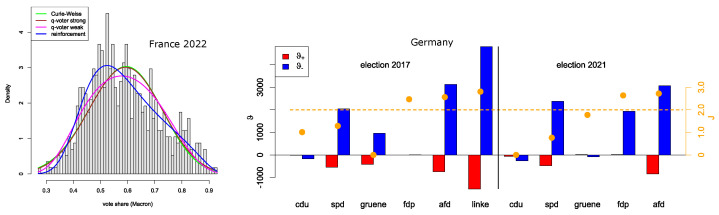
Right: Histogram of the vote shares for the presidential elections in France (2022), together with the probability density for the four models. Left: Histogram of $\vartheta_{\pm }$ and *J* (together with critical level, orange) for the parties in 2017, 2021, which hold more than 5% of the votes (threshold), apart from the CSU, which is a Bavarian local.

German data: As we have a proportional electoral system, the dichotomous model requires adaptation—for each party, we distinguish between the votes in favor of this party versus the votes for all other parties. In Figure 3, we find the reinforcement parameters together with the coupling *J* of the Curie–Weiss model for those parties in the 2017 and 2021 elections that did receive at least 5% of the votes (where we did disregard the CSU, as this is a Bavarian local party that only stands for election in few election districts). If we focus on the best models (lowest AIC), these models indeed are able to meet the empirical vote share distributions quite well (Kolmogorov–Smirnov test), with only one exception: the left-wing party Left Party (die linke) in the 2017 election. Due to historical reasons, this party performs very differently in the federal states coming from the former East and West Germany, respectively. Though these historical effects can also be understood to be based on opinion dynamics and in/out-group behavior, all models have difficulties in capturing this data structure. The assumption of a homogeneously mixed population may no longer be appropriate; instead, a two-island model would capture the communication structure better. Also, the (relatively recent) right-wing party AfD, which also performs rather differently in the two regions (former East/West Germany), poses a problem for all models but the reinforcement model, at least according to the Kolmogorov–Smirnov test. However, the difference in the two regions is less pronounced than in the case of the Left Party, which might also be the reason why the reinforcement model is still able to handle the vote share data of the AfD.

**Figure 4:**
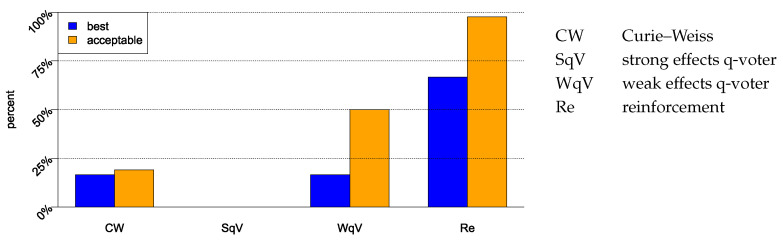
Performance of the four models. For the 42 elections we consider in the present paper, we indicate the percentage for which the models performed best according to the AIC (yellow) and acceptable (AIC has a maximum distance of 2 to the best model).

Summary of the estimations: In almost all elections, at least some of the models describe the empirical data adequately (according to the Kolmogorov–Smirnov test). If we compare the performance of the models according to the AIC, we find that the weak effects models outperform the strong effects models, and the weak effects reinforcement model is superior to the weak effects q-voter model (Figure 4). It is interesting that although the structures of the weak effects models are very similar, we nevertheless find a difference in their suitability for practical applications.

We should keep in mind that we work with aggregated data (only the outcome of election in election districts that typically have a population of hundreds of thousands of individuals). We might consider the model parameters as a reduction of the data complexity to a low-dimensional parameter space, which allows us to better interpret the data. As the Kolmogorov–Smirnov test indicates the appropriateness of the models, we can be confident that the models capture at least some fundamental structure in the data. In that, the interpretation of the parameters suggested by the models will be appropriate.

### Political Science Interpretations

United States: Looking at the parameters and measuring the actual strength of reinforcement in the reinforcement model, we can see that since the year 2000, there has been a continuous trend for a much more polarized voting behavior in the United States. For the voters of the Republican Party, the switch could already be seen in 2008 with the election of Barack Obama, and it continued in his re-election, while it became dominant in 2016 and 2020 when Donald Trump became candidate of the Republican Party. The voters for the Democratic Party clearly also changed from open- to closed-mindedness in the political realm. In fact, what we can see is that during those 20 years, the political discourse became so polarized in the US public arena that now both the parties and their voters are becoming clearly more and more separated from each other. For example, Binder [42] shows that the frequency of legislative gridlock has risen since the 1990s, and other studies show that polarization in the US citizenry is not going down [43].

Great Britain: Due to the electoral system (first-past-the-post) and the governmental system (parliamentary), it is clearly useful for the two dominant parties—the Labour Party on the left and the Conservative Party (Tories) on the right—to keep a certain or even a high degree of polarization so that they can form the government on their own. The bars of the reinforcement model show this clearly for Labour for almost all elections since 1945, but for the Tories, this strategy started only in the 1970s and became very dominant since the second election in 1974 and the Thatcher years, 1979–1990. Additionally, we can see that in 2019, the reinforcement parameters had not been so strong. This might be the result of the Brexit decision in 2016 and its political aftermath in which the Conservatives had become the party of the Leavers (in support of Brexit) and the Remainers have split between Labour and the Liberal Democrats [44].

**Figure A3:**
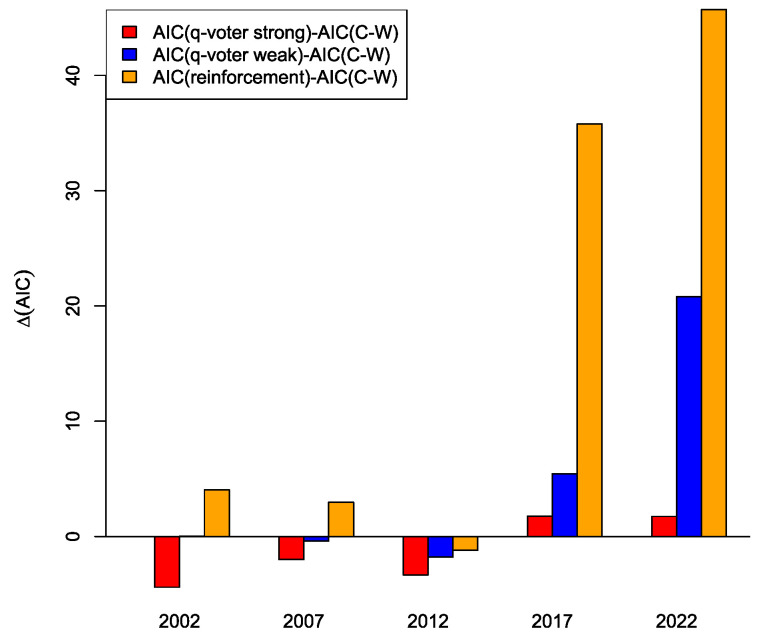
Comparison of the models’ AICs for France.

France: The French Party System has undergone a political change in the last few years since 2017, so some already argue that we may see the rise of a new French Party System [45]. In 2017, both presidential candidates of the traditional left-wing (Parti Socialiste) and right-wing parties (Les Républicains) were disqualified in the first round of the presidential elections [46]. And this happened again in 2022, when both Emmanuel Macron of the centrist “La République En Marche” and Marine Le Pen of the new-named right-wing populist “Rassemblement National” were, for the second time, the political opponents in the second round of the election [47]. That said, we can see in Figure A3 for the previous elections that the models did not explain so much for the second round.

Germany: Here, we can see that the right-wing populist party Alternative for Germany (AfD) was able, in both elections, to create their own space for resonating with their voters. In 2017, both the Social Democrats (SPD), the Left Party, and the Greens had been able to mobilize their voters against the AfD, but in 2021, it was the SPD and the liberal party (FDP). Compared to the left parties, the FDP tried to position itself as the party of Freedom, where some parts of the party also raised their critical voice against the political means of the former government of the two conservative parties CDU and CSU and the Social Democrats used during the Covid-19 pandemic. This can be observed in their parliamentary work, where they used so called “Kleine Anfragen” to question the government parties the most compared to all the other remaining opposition parties in the German Bundestag [48]. Therefore, they had been a competitor both to the AfD and the other left and center parties and also gained a lot of support by young voters [49].

## 6. Discussion of the Findings and Their Interpretation in Political Sciences

In the present paper, we first discussed the connection between Potts models and stochastic opinion models, both for well mixing populations. In particular, we did provide an alternative approach to the q-voter model as a natural extension of the zealot model in the view of Glauber dynamics. Consequently, motivated by similar constructions in population genetics, we introduced a strong and also a particularly weak effects continuum limit. While the strong continuum limit is generically possible (and locally approximates a normal distribution), the weak effects limit requires additional structure. In that, the Curie–Weiss model only allows for the strong limit, while the q-voter model has both limits. Afterwards, we additionally introduced the reinforcement model, which has its foundation not in the Potts machinery, but is derived based on considerations of the impact of several kinds of cognitive biases on communication, especially on the resulting in/out-group communication strategies. Also, that model allows for both continuum limits, where it turned out that the weak q-voter and the weak reinforcement model are very similar in their mathematical structure. Basically, both are an adaptation of the beta distribution, which is the well-known weak effects limit of the zealot (or Moran) model [33]. We also found that only models that are based on the voter model allow for a weak effects limit, which indicates that these models are in some sense special.

After these theoretical considerations, we turned to test the models based on election data. Herein, we used each election district as an i.i.d. repetition of the election, neglecting social co-factors, which vary between election districts, as well as spatial factors. We also assume the opinion process to be approximately in equilibrium such that the stationary distribution is an adequate description of the data; in case of a large shift in the vote share of a candidate or party, this assumption can also be called into question. Though the approach was simple, we mostly found that the models meet the data quite well, where the weak models performed better than the strong models, and the weak reinforcement model outperformed the weak q-voter model. It is interesting that the weak effects models seem to be better suited to describe the data appropriately. The comparison of models and data is always challenging, but it also seems that population genetics, where the weak effects such as weak selection are often used, rather supports that these kind of models are well suited for real world applications [50]. The background could be that striking and immediately disruptive events are rare. Most stimuli are weak and require time to unfold their effect. If this observation is correct, weak effects models with their slow time scale might indeed be a better description of reality than strong effects models with a fast time scale. As a practical consequence, we propose to use rather weak effects opinion models than strong effects models in empirical studies, which also has the advantage of not needing to choose an appropriate population size, which is a well-known problem in itself [26,33]. Though the models allowing for a weak effects limit are rather special, they seem to be a powerful description of reality and still have sufficient flexibility to address different mechanisms and different real world (electorate) systems.

Elections are at the center stage of modern representative democracies. Correspondingly, research on elections and attempts to explain the formation of their results are also central. Prominent and established approaches use statistical data concerning the social characteristics of voters to determine their voting behavior (e.g., [51]). But, there is an ongoing debate among scholars that the correlation between social characteristics and voting behavior has diminished over the last few decades (e.g., [52,53,54]). Additionally, party membership is also in decline, which also has consequences for voter turnout and voting behavior (e.g., [55,56]). As Clarke et al. [57] bluntly declared, for understanding electoral choice, one has to look elsewhere. It is not that the classic approaches have lost all their explanatory power, but it makes sense to look for explanations that are less context-dependent.

By focusing on the dynamics of opinion formation preceding the act of voting, the models discussed in this paper promise insights both into the empirical explanation of elections as such, as well as important aspects of the theory of democracy.

Our leading assumption, ensuring a larger independence of specific social contexts, is opinion formation via frequently contacting social sources of information, constituting a ubiquitous mechanism of collective decision-making. For sure, this assumption also holds for elections. The sources of information here may be real persons or media of all kinds. Pamphlets, newspapers, magazines, radio, TV, and the diversity of social media have accompanied political discussion since the early modern period. Albeit the basic mechanism is taken to be the same everywhere, its effects may be modulated by the impact of or the interaction with other mechanisms of other sections within an “organized complexity”. Electoral systems in their specific forms are nested within the broader construction of a political system. The effects of opinion formation processes concerning elections are shaped by specific institutional settings as well as the political culture of the respective countries.

The model’s reduction to only two opinions may look like too crude a simplification. But it is not as implausible as it may appear at first glance. As Denver and Johns [58] stress, when preparing their decisions, voters don’t “sit down before an election to comb through the parties’ manifestos and make detailed calculations of the costs and benefits of voting for each party. (…) Such a process is neither realistic nor particularly rational ([58] p. 294)”. Rather, voters base their decision on just a few subjects dominating the discussion. “Issue Voting” and “Valence Voting” denominate the approaches based on that assumption. Where Issue Voting stresses the main issues of the election campaign like economic questions or social policy, Valence Voting focuses, for example, on the performance of the incumbent government. “Neither involves complex calculations; indeed, the simpler versions of both approaches have fared better when confronted with the empirical evidence (ibid.)”. And both aspects fare better than explaining results with respect to voters’ social characteristics. Therefore, looking at opinion and opinion formation in this simple form offers a promising starting point to delve deeper into campaigning and voting.

In this view, the models in their present form may be interpreted as to suppose a stage of the election process, where the main issues are already settled. From here, the model may be enhanced by stepwise nesting the basic opinion formation process within a whole set of similar modeled ones. The determination of the salient issues and valences may be the next step. The party that succeeds in putting its topics in place may be at an advantage. A variant of the Issue Voting approach stresses that it is not only the preferences on the specific topics of the very election of today that affect the voter’s decision, but general values and principles ([58] p. 295). We can think of processes concerning ideological backgrounds running on a larger time scale spanning across two or more elections, affecting the probability with which a voter makes up his mind. Another perspective would look at the developments within the zealots in particular. This would mean looking at the development of party programs and strategies, also in the form of opinion formation among party members. The possibilities are manifold.

The reinforcement model in particular also highlights important aspects concerning the theory of democracies. Especially in the liberal tradition of democracy, it is a common view to interpret campaigning and elections as a market analogous competition, where votes are exchanged for programs and personnel (see [59]). Competition appears as a form of regulated and limited conflict. The opponent’s purpose is not to harm the antagonist, but only to be better. The idea behind this is that the aspiration to trump the adversary leads to the advancement of a common good, qualitatively better or cheaper products and processes in economy, better theories and methods in science, and better programs and personnel in politics.

The zealot model, as a predecessor of the reinforcement model, was used before in economics for market analysis [60]. But behind this application lies a model designed to explain foraging processes of ant colonies [61]. It describes a form of collective information processing against an uncertain environment. Time and again the colony has to leave an established feeding ground and look for another in time. It is inspiring to see the similarities. Political communities also have to alter their processes and organization because of altering circumstances, for example, transforming their way of living to a more sustainable way. In this way, an open political process, defining problems and looking for solutions, is a form of collective information processing, too. This idea was emphasized by Karl Popper [62], for example, and further developed by John Dryzek [63]. It is not implausible to assume that a part of the success of democracies in general is their dealing with the world’s shakiness in an analogous way to modern science.

Whatever makes the workers of the ant colony change their paths, the driving force behind the parliamentary process, at least from the perspective of the liberal standard model of democracy, is party competition. Since party competition is itself affected by special interests and personal ambition of politicians, democracies need additional features to balance these forces and bring the wanted effects of the competition to the fore. This has been part of the considerations from Harrington [64] to Tocqueville [65] to Dewey [66] but shall not be the point here.

What the reinforcement model enables us to see is the possible polarization between the opponents. It is important to note that polarization indicating a higher grade of conflict is not a problem per se, as higher grades of conflict are not as well. As sociologist Lewis Coser (1967) [67] argued for, conflicts in the first place point to societal problems within a society urging to deal with their causes. If the community is productively addressing that challenge, society reintegrates on a new level. We could see such effects, for example, in the course of the environmental movement in the 1970s to 1990s in Germany. The polarization on the side of the Greens was high in the beginning when they cracked open the consensus of the established parties on the use of nuclear power, and became lower again when environmental issues were successfully established on the agenda.

Polarization may become problematic when the reinforcement effects are strong on both sides of the debate. Conflicts can be disruptive too. American philosopher of law Ronald Dworkin was already asking in 2006 [68], looking at the polarization in the US “Is Democracy possible here?”. The polarization in the US has not been in decline since then (Pew Research Center 2022, [43]). Polarization may also become problematic when actors show no tendency to consent or compromise, enabling reintegration. This appears to be the case with the populist movements and parties of the last decade. On the other hand, party polarization may generate stronger party attachments, which could also be a desirable strategy for political competitors ([69] p. 350). And here is probably the point where political scientists (at least at the moment) have to reach out for other methods than mathematical modeling, too. Qualitative analysis of texts or focus groups may be an appropriate means here. However, it should be ascertained that the reinforcement model supplies us with a strong indicator concerning an important variable of political processes. And since the claim that a society is polarized is also often used in an alarmist way, impeding compromise, the more it is helpful to have this indicator. It should be ascertained further that because of its context independence, the model will be useful when we look not only at well-established democracies of the West, but also at young ones or democracies in other world regions.

## Figures and Tables

**Table 1:** Design of the study: data set used.

	US	UK	France	Germany
Government system	presidential	parliamentary	semi-presidential	parliamentary
Electoral system	Plurality/majority: single-member districts, first-past-the-post, relative majority	Plurality/majority: single-member districts, first-past-the-post, relative majority	Plurality/majority: two-round system, absolute majority	Mixed: mixed member proportional
Election years covered in the analysis	2000–2020 (6 presidential elections)	1945–2019 (20 parliamentary elections)	2002–2022 (5 presidential elections, second round)	2017–2021 (2 parliamentary elections)

**Figure A2:**
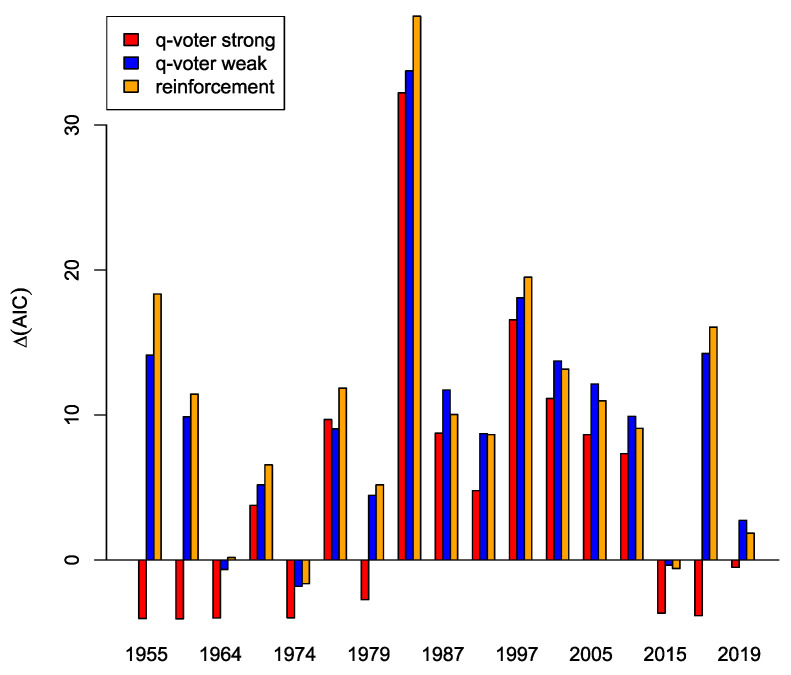
Comparison of the models’ AICs for the UK.

**Figure A4:**
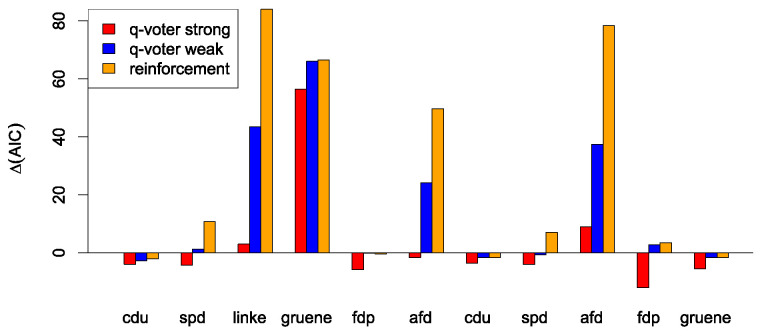
Comparison of the models’ AICs for Germany.

## Data Availability

United States Data. https://dataverse.harvard.edu/dataset.xhtml?persistentId=doi:10.7910/DVN/VOQCHQ All data from the US were accessed on 24 June 2021. France data. 2002–2012: https://www.data.gouv.fr/fr/posts/les-donnees-des-elections/ 2017: https://www.data.gouv.fr/fr/datasets/election-presidentielle-des-23-avril-et-7-mai-2017-resultats-definitifs-du-2nd-tour/ 2022: https://www.data.gouv.fr/fr/datasets/election-presidentielle-des-10-et-24-avril-2022-resultats-definitifs-du-2nd-tour/ All France data were accessed on 8 December 2023. United Kingdom data. https://commonslibrary.parliament.uk/research-briefings/cbp-8647 All UK data were accessed on 5 November 2021. German data. https://www.bundeswahlleiter.de/en/bundeswahlleiter.html The German data for the 2017 election were accessed on 8 July 2018; that for the 2021 election on 5 October 2022.

## Appendix A. Data Analysis

The maximum likelihood estimates were performed in R [70], based on the optimizer nlm. In the case of the Curie–Weiss and the strong q-voter model, all parameters were optimized in one run. The weak effects models have two parameters related to the zealots ($N^{\pm }$ and $\eta^{\pm }$, respectively), and two parameters related to ingroup/outgroup communication structures ($\lambda_{\pm }$, $\kappa_{\pm }$, and $\vartheta_{\pm }$). Here, we used an EM-like algorithm by alternately optimizing the parameters for the zealots and holding the in/outgroup parameters, and then optimizing the parameters for the in/outgroup while holding the zealot parameters until the vector of parameters became approximately constant.

The Kolmogorov–Smirnov test was performed to test for the appropriateness of the current model, and the AIC was determined in order to compare the models. The columns of the tables are to be read specifically for the model, e.g., the $J/\eta^{+}/N^{+}$ column holds *J* in case of the Curie–Weiss model, $\eta^{+}$ for the strong q-voter model, and $N^{+}$ for the weak q-voter model and reinforcement model.

In the resulting tables, we mark the models that have the minimal AIC (best performing models) for the corresponding elections in yellow. Of course, if the AIC of two models is close, it is not sensible to dismiss one of the two models. Burnham et al. [71] suggest that models that have a difference of at most two do perform comparably. These models are marked in gray. Additionally, we provide a graphic for the differences in the AICs. Here, we use for each given election the AIC of the Curie–Weiss model as a reference and provide

ΔAIC=AIC other model−AIC Curie−Weiss.

That is, if this value is positive, the “other model” performs better than the Curie–Weiss model; if it is negative, the Curie–Weiss model performs better. Moreover, if ΔAIC is, e.g., larger for the weak q-voter model than for the reinforcement model, then the weak q-voter model performs better than the reinforcement model.

### Appendix A.1. United States Data

US presidential elections in the years 2000–2020 were analyzed, where we only considered Democrats and Republicans and the vote share of the Democrats (e.g., votes for Democrats/votes for Democrats or Republicans) and dismiss the votes for any other candidate. The performance of the AICs for the models is shown in Figure A1.

**Year**	**Model**	**J/$\eta^{+}$/$N^{+}$**	**h/$\eta^{-}$/$N^{-}$**	**$\lambda_{+}$/$\kappa_{+}$/$\vartheta_{+}$**	**$\lambda_{-}$/$\kappa_{-}$/$\vartheta_{-}$**	**N**	**AIC**	$p_{\mathrm{KS}}$
2000	Curie–Weiss	0.015	−0.38	−	−	15.1	−4299.2	0.04
2000	q-voter strong	7	19.5	−2.7	−12.7	12.2	−4295.5	0.046
2000	q-voter weak	3.7	0.00031	−3.2	−22.1	−	−4296.3	0.023
2000	reinforcement	3	6.9 $\times \;10^{-6}$	−15.5	−39.6	−	−4316.3	0.1
2004	Curie–Weiss	1.3	−0.2	−	−	31.8	−4150.6	0.39
2004	q-voter strong	31.9	50.2	36.6	69.4	31.5	−4146.4	0.43
2004	q-voter weak	6.8	2.8 $\times \;10^{-6}$	7.5	−21.2	−	−4156.4	0.29
2004	reinforcement	4.4	0.12	−3.5	−34.3	−	−4172.4	0.48
2008	Curie–Weiss	1.5	−0.11	−	−	34.2	−3489.9	0.31
2008	q-voter strong	15.8	20.3	23.3	32.7	35	−3485.6	0.23
2008	q-voter weak	7.9	0.04	11.1	−17.3	−	−3511.3	0.49
2008	reinforcement	5	1.3	1.7	−22.4	−	−3517.7	0.28
2012	Curie–Weiss	1.6	−0.13	−	−	31.7	−3074.7	0.15
2012	q-voter strong	22	25.2	35.5	42.8	31.8	−3070.6	0.19
2012	q-voter weak	6.5	0.018	10.5	−14.7	−	−3101.3	0.3
2012	reinforcement	4.3	0.92	4.2	−19.3	−	−3115.6	0.88
2016	Curie–Weiss	2.1	−0.085	−	−	56.8	−3167.7	0.71
2016	q-voter strong	4.9	8	10.1	19.3	58.2	−3162.5	0.79
2016	q-voter weak	9.3	2.4	22.3	−5	−	−3168.7	0.3
2016	reinforcement	6.4	4.1	26.7	6.5	−	−3173.9	0.59
2020	Curie–Weiss	2.1	−0.084	−	−	55.7	−3066	0.51
2020	q-voter strong	13.8	25.3	25.7	59.7	56.6	−3061.8	0.59
2020	q-voter weak	9.2	3.5	21.5	−2	−	−3072.5	0.84
2020	reinforcement	6.5	5.2	26.9	11.3	−	−3071.1	0.69

### Appendix A.2. UK Data

We investigated the vote share of the conservative among the conservative and Labour votes. The performance of the AICs for the models is shown in Figure A2.

**Year**	**Model**	**J/$\eta^{+}$/$N^{+}$**	**h/$\eta^{-}$/$N^{-}$**	$\lambda_{+}$/$\kappa_{+}$/$\vartheta_{+}$	$\lambda_{-}$/$\kappa_{-}$/$\vartheta_{-}$	**N**	**AIC**	$p_{\mathrm{KS}}$
1945	Curie–Weiss	3.3 $\times \;10^{-8}$	−0.26	−	−	12.1	−593.4	0.86
1945	q-voter strong	7.6	0.67	−6.1	−0.56	9.5	−589.6	0.84
1945	q-voter weak	1.1	11.2	−13.7	10.6	−	−592.6	0.16
1945	reinforcement	3.3	11.7	−12.8	16.9	−	−596.7	0.64
1950	Curie–Weiss	7.1 $\times \;10^{-8}$	−0.039	−	−	12.2	−628.5	0.64
1950	q-voter strong	4.2	4.1	−24	−23	3.7	−624.9	0.61
1950	q-voter weak	0.098	21.3	−21.7	40.3	−	−643.4	0.98
1950	reinforcement	2.8	15	−16.5	35.1	−	−646.2	0.96
1951	Curie–Weiss	6.4 $\times \;10^{-7}$	0.013	−	−	13.1	−652.9	0.34
1951	q-voter strong	2.6	−0.0066	−9.4	−0.68	6.5	−651.4	0.45
1951	q-voter weak	3.3 $\times \;10^{-8}$	29.3	−26.1	60.6	−	−666.4	0.92
1951	reinforcement	2.6	14.4	−19.3	32.2	−	−669.4	0.94
1955	Curie–Weiss	1.5	0.018	−	−	43.8	−715.8	0.65
1955	q-voter strong	17.9	16.5	28.5	25.5	43.4	−711.8	0.74
1955	q-voter weak	1.7	17.7	−16.5	31.9	−	−730	0.96
1955	reinforcement	5.3	16.8	−3.5	47.4	−	−734.2	0.87
1959	Curie–Weiss	1.7	0.022	−	−	63.2	−699.5	0.47
1959	q-voter strong	27.2	24	49.4	41.4	61.6	−695.4	0.52
1959	q-voter weak	3.3	17.9	−11.8	33.4	−	−709.4	0.82
1959	reinforcement	5.7	14	0.018	38.8	−	−710.9	0.88
1964	Curie–Weiss	1.1	−0.011	−	−	22.4	−639	0.72
1964	q-voter strong	9.6	9.5	11.3	11.1	21.9	−635	0.75
1964	q-voter weak	2	5.5	−9.1	0.89	−	−638.4	0.87
1964	reinforcement	3.1	5.3	−11.2	−1.9	−	−639.2	0.95

**Year**	**Model**	**J/$\eta^{+}$/$N^{+}$**	**h/$\eta^{-}$/$N^{-}$**	$\lambda_{+}$/$\kappa_{+}$/$\vartheta_{+}$	$\lambda_{-}$/$\kappa_{-}$/$\vartheta_{-}$	**N**	**AIC**	$p_{\mathrm{KS}}$
1970	Curie–Weiss	0.66	0.017	−	−	15.1	−625.1	0.41
1970	q-voter strong	−0.62	0.56	0.73	2.2	67.9	−628.9	0.96
1970	q-voter weak	0.35	10	−15.3	13.8	−	−630.3	0.91
1970	reinforcement	1.9	6.5	−17.1	3.8	−	−631.7	0.89
1974	Curie–Weiss	1.9	0.0039	−	−	50.5	−497.5	0.7
1974	q-voter strong	5.7	5.6	11.6	11.2	50.6	−493.5	0.69
1974	q-voter weak	8.2	7.7	10	8.8	−	−495.7	0.65
1974	reinforcement	7.3	6.6	16.4	13.7	−	−495.9	0.74
1974	Curie–Weiss	1.9	−0.018	−	−	51.7	−450.8	0.15
1974	q-voter strong	0.042	0.22	1	1.5	178.1	−460.5	0.88
1974	q-voter weak	7.1	14.2	6.8	26.8	−	−459.8	0.34
1974	reinforcement	7.1	13.2	20.5	45.6	−	−462.6	0.89
1979	Curie–Weiss	1.9	0.021	−	−	57.5	−473.5	0.31
1979	q-voter strong	10.4	3.8	25.4	6.7	59.7	−470.8	0.37
1979	q-voter weak	18.4	7.8	37	7.1	−	−478	0.63
1979	reinforcement	12.6	7.7	42.2	23.1	−	−478.7	0.65
1983	Curie–Weiss	2	0.072	−	−	32.8	−327.7	0.044
1983	q-voter strong	1.4	0.04	5	0.81	108.1	−360	0.54
1983	q-voter weak	22.2	5.3	51.4	3.8	−	−361.5	0.51
1983	reinforcement	14.2	5.6	56.3	22	−	−365.3	0.67
1987	Curie–Weiss	2.1	0.029	−	−	51.9	−293.6	0.21
1987	q-voter strong	0.016	0.011	1	1	692.9	−302.4	0.61
1987	q-voter weak	11.1	8.6	23.6	17.9	−	−305.4	0.55
1987	reinforcement	8.6	6.7	36.1	30.6	−	−303.7	0.57
1992	Curie–Weiss	2.1	0.017	−	−	52.5	−309.1	0.2
1992	q-voter strong	0.2	0.099	1.5	1.2	114.1	−313.9	0.44
1992	q-voter weak	12.3	8.1	25.8	14.3	−	−317.8	0.47
1992	reinforcement	9.2	6.7	36.3	26.8	−	−317.7	0.6
1997	Curie–Weiss	2	−0.056	−	−	40.4	−376	0.34
1997	q-voter strong	0.07	1.8	0.83	6	96.3	−392.6	0.52
1997	q-voter weak	6.2	16	7.3	33.8	−	−394.1	0.9
1997	reinforcement	6.2	13.1	22.8	49.1	−	−395.5	0.54
2001	Curie–Weiss	2	−0.044	−	−	44.6	−363.2	0.48
2001	q-voter strong	0.039	0.15	1	1.4	183.1	−374.3	0.22
2001	q-voter weak	6.7	15.2	9.1	32.2	−	−376.9	0.37
2001	reinforcement	6.2	11.3	23.5	42.3	−	−376.3	0.22
2005	Curie–Weiss	2.1	−0.018	−	−	56.5	−316.5	0.2
2005	q-voter strong	0.052	0.13	1.1	1.3	173.7	−325.2	0.17
2005	q-voter weak	7.9	16.6	12.2	37.1	−	−328.7	0.21
2005	reinforcement	7.1	11	28.9	44.9	−	−327.5	0.18
2010	Curie–Weiss	2.1	0.021	−	−	39.4	−211.1	0.061
2010	q-voter strong	0.0093	0.0073	1	1	709.6	−218.4	0.15
2010	q-voter weak	7.7	6.1	15.6	11.9	−	−221	0.12
2010	reinforcement	6.2	4.9	25	20.4	−	−220.1	0.17
2015	Curie–Weiss	2.2	0.0076	−	−	83.4	−307.9	0.71
2015	q-voter strong	1.7	1.6	4.9	4.4	86.9	−304.2	0.71
2015	q-voter weak	13.8	14.1	30.8	32.4	−	−307.5	0.79
2015	reinforcement	10.5	10.4	49.9	50.8	−	−307.3	0.79
2017	Curie–Weiss	2	0.0039	−	−	76	−416.3	0.47
2017	q-voter strong	4.9	5.1	10.8	11.6	77	−412.5	0.5
2017	q-voter weak	8.7	17.8	10.9	38.8	−	−430.6	0.45
2017	reinforcement	8.2	14.4	29.4	57.7	−	−432.4	0.61
2019	Curie–Weiss	2.1	0.036	−	−	45.5	−314.8	0.77
2019	q-voter strong	0.043	0.065	1.1	1.2	143.9	−314.2	0.57
2019	q-voter weak	6.3	7.6	9.2	15.5	−	−317.5	0.8
2019	reinforcement	5.3	5.9	17.1	22.9	−	−316.6	0.77

### Appendix A.3. France Data

This is the second round of the presidential election, and we considered the vote share of the winning candidate. The performance of the AICs for the models is shown in Figure A3.

**Year**	**Model**	**J/$\eta^{+}$/$N^{+}$**	**h/$\eta^{-}$/$N^{-}$**	$\lambda_{+}$/$\kappa_{+}$/$\vartheta_{+}$	$\lambda_{-}$/$\kappa_{-}$/$\vartheta_{-}$	**N**	**AIC**	$p_{\mathrm{KS}}$
2002	Curie–Weiss	0.95	0.98	−	−	58	−1672.5	0.11
2002	q-voter strong	35.5	6	14	2	47	−1668.1	0.099
2002	q-voter weak	23.5	5	−56.5	−7	−	−1672.5	0.16
2002	reinforcement	533	9	1275.5	−452.7	−	−1676.5	0.32
2007	Curie–Weiss	0.097	0.11	−	−	41.8	−1297.7	0.58
2007	q-voter strong	3.2	2.9	−280.7	−241.6	1.1	−1295.7	0.66
2007	q-voter weak	41.1	5.3 $\times \;10^{-5}$	56.2	−57	−	−1297.3	0.48
2007	reinforcement	23.2	6.1 $\times \;10^{-5}$	−0.32	−86.2	−	−1300.7	0.29
2012	Curie–Weiss	1.4 $\times \;10^{-7}$	0.082	−	−	29.9	−1138	0.2
2012	q-voter strong	2.8	2.6	−162.4	−145.5	1.2	−1134.7	0.23
2012	q-voter weak	0.006	0.00031	−33.9	−30.4	−	−1136.2	0.22
2012	reinforcement	0.54	0.0014	−66.8	−64.1	−	−1136.8	0.24
2017	Curie–Weiss	1.2 $\times \;10^{-7}$	0.74	−	−	14.3	−819.2	0.18
2017	q-voter strong	8.1	4.5	−817	−343.8	0.41	−821	0.25
2017	q-voter weak	0.064	1	−27.6	−8.8	−	−824.7	0.24
2017	reinforcement	109.6	6.3	325.2	−61.2	−	−855	0.95
2022	Curie–Weiss	1.5 $\times \;10^{-9}$	0.38	−	−	13.4	−718.8	0.0077
2022	q-voter strong	0.009	0.029	−206.6	−129.5	0.085	−720.5	0.003
2022	q-voter weak	85.1	0.11	192	−49.2	−	−739.6	0.097
2022	reinforcement	65.6	7.4	211.9	−17.2	−	−764.5	0.96

### Appendix A.4. German Data

We include all parties that did reach a vote share of at least 5% except the CSU, which is a local party and thus only stands for election in a few districts. Note that “die linke” is present in the parliament of 2021, though this party did not reach 5%, and in that, we did exclude this party in 2021. In order to fit our dichotomy model, we focused on the vote share of the focal party, essentially distinguishing between supporters of this party and supporters of any other party. The performance of the AICs for the models is shown in Figure A4.

**Year**	**Model**	**Party**	**J/$\eta^{+}$/$N^{+}$**	**h/$\eta^{-}$/$N^{-}$**	$\lambda_{+}$/$\kappa_{+}$/$\vartheta_{+}$	$\lambda_{-}$/$\kappa_{-}$/$\vartheta_{-}$	**N**	**AIC**	$p_{\mathrm{KS}}$
2017	Curie–Weiss	cdu	1	−0.41	−	−	123.1	−745.2	0.81
2017	q-voter strong	cdu	22.6	48.9	19.8	57.9	123.4	−741.2	0.81
2017	q-voter weak	cdu	14	26.7	−15	−56.3	−	−742.5	0.87
2017	reinforcement	cdu	14.4	9.4 $\times \;10^{-5}$	−14.7	−173	−	−743.1	0.83
2017	Curie–Weiss	spd	1.3	−0.63	−	−	68.7	−814.1	0.23
2017	q-voter strong	spd	7.2	31.3	5	33.1	55.5	−809.8	0.17
2017	q-voter weak	spd	10.1	0.27	18.9	−80.6	−	−815.3	0.18
2017	reinforcement	spd	19.6	752.9	−545.5	2039.8	−	−824.8	0.4
2017	Curie–Weiss	linke	2.8	−0.11	−	−	81.9	−1068.6	3.6 $\times \;10^{-8}$
2017	q-voter strong	linke	0.06	20.5	0.6	82	108.6	−1071.6	2.7 $\times \;10^{-7}$
2017	q-voter weak	linke	9.1	2.7	42.8	9.3	−	−1112.1	5.7 $\times \;10^{-7}$
2017	reinforcement	linke	14.2	1800.1	−1509.8	4806	−	−1152.6	6 $\times \;10^{-5}$
2017	Curie–Weiss	gruene	2 $\times \;10^{-6}$	−2.8	−	−	19.2	−1047.3	2.6 $\times \;10^{-6}$
2017	q-voter strong	gruene	0.95	45.2	0.72	12.5	50.8	−1103.8	0.26
2017	q-voter weak	gruene	4.7	0.063	15.3	−164.1	−	−1113.4	0.7
2017	reinforcement	gruene	4.4	472.5	−421.4	961.3	−	−1113.8	0.71
2017	Curie–Weiss	fdp	2.5	−0.21	−	−	244.3	−1320.7	0.5
2017	q-voter strong	fdp	11.9	116.1	1.3	8.4	131.5	−1314.9	0.34
2017	q-voter weak	fdp	17.2	5.4	53.7	−308.8	−	−1320.5	0.57
2017	reinforcement	fdp	14	117.1	0.14	1.1	−	−1320.2	0.57
2017	Curie–Weiss	afd	2.6	−0.088	−	−	110	−985.3	0.0082
2017	q-voter strong	afd	1.1	23	2.2	86.7	117.5	−983.7	0.011
2017	q-voter weak	afd	14.5	8.9	54.5	23.7	−	−1009.4	0.094
2017	reinforcement	afd	22.4	1081.9	−747.5	3127.1	−	−1035	0.77
2021	Curie–Weiss	cdu	2.6 $\times \;10^{-7}$	−1.3	−	−	64	−778.3	0.6
2021	q-voter strong	cdu	16.6	53	−40	−215.2	30.8	−774.6	0.75
2021	q-voter weak	cdu	1.8	8.8	−31	−156.9	−	−776.6	0.76
2021	reinforcement	cdu	3.5	0.8	−60.7	−261.1	−	−776.6	0.75
2021	Curie–Weiss	spd	0.76	−0.7	−	−	72.8	−829.6	0.74
2021	q-voter strong	spd	13.1	38.5	5.3	21.2	63	−825.5	0.74
2021	q-voter weak	spd	13.1	1.2	14	−97.2	−	−828.9	0.74
2021	reinforcement	spd	38.7	827.5	−477	2376.9	−	−836.6	0.93

**Year**	**Model**	**Party**	**J/$\eta^{+}$/$N^{+}$**	**h/$\eta^{-}$/$N^{-}$**	$\lambda_{+}$/$\kappa_{+}$/$\vartheta_{+}$	$\lambda_{-}$/$\kappa_{-}$/$\vartheta_{-}$	**N**	**AIC**	$p_{\mathrm{KS}}$
2021	Curie–Weiss	afd	2.7	−0.12	−	−	71.9	−971.4	1.3 $\times \;10^{-5}$
2021	q-voter strong	afd	0.044	14.9	0.59	56.4	122.8	−980.4	7.7 $\times \;10^{-5}$
2021	q-voter weak	afd	8.6	4.1	37.3	11	−	−1008.8	0.0014
2021	reinforcement	afd	13.7	1092.2	−841	3064.4	−	−1049.7	0.21
2021	Curie–Weiss	fdp	2.6	−0.032	−	−	430.3	−1424.6	0.37
2021	q-voter strong	fdp	19.7	170.3	1.5	11.5	198.3	−1412.6	0.15
2021	q-voter weak	fdp	40.9	254	74.7	703.9	−	−1427.3	0.64
2021	reinforcement	fdp	43.3	522.6	19	1933.9	−	−1428	0.79
2021	Curie–Weiss	gruene	1.8	−0.61	−	−	52.2	−813.8	0.18
2021	q-voter strong	gruene	1.2	24.7	0.98	21.2	36.2	−808.2	0.081
2021	q-voter weak	gruene	3.8	22.1	−2.1	−21.3	−	−812.1	0.13
2021	reinforcement	gruene	3.8	0.3	13.9	−87.8	−	−812.2	0.13
